# Transcriptome-wide high-throughput deep m^6^A-seq reveals unique differential m^6^A methylation patterns between three organs in *Arabidopsis thaliana*

**DOI:** 10.1186/s13059-015-0839-2

**Published:** 2015-12-14

**Authors:** Yizhen Wan, Kai Tang, Dayong Zhang, Shaojun Xie, Xiaohong Zhu, Zegang Wang, Zhaobo Lang

**Affiliations:** State Key Lab Laboratory of Crop Stress Biology for Arid Areas, College of Horticulture, College of Life Sciences, Northwest A&F University, Yangling, Shaanxi 712100 China; Department of Horticulture and Landscape Architecture, Purdue University, West Lafayette, IN 47907 USA; Shanghai Center for Plant Stress Biology, Shanghai Institutes for Biological Sciences, Chinese Academy of Sciences, Shanghai, 200032 China; Institute of Biotechnology, Jiangsu Academy of Agricultural Sciences, Nanjing, 210014 China; College of Bioscience and Biotechnology, Yangzhou University, Yangzhou, 225009 China

**Keywords:** *N*^6^-methyladenosine, m^6^A mapping, Transcriptome-wide patterns, RNA differential methylation, Transposable element

## Abstract

**Background:**

m^6^A is a ubiquitous RNA modification in eukaryotes. Transcriptome-wide m^6^A patterns in *Arabidopsis* have been assayed recently. However, differential m^6^A patterns between organs have not been well characterized.

**Results:**

Over two-third of the transcripts in *Arabidopsis* are modified by m^6^A. In contrast to a recent observation of m^6^A enrichment in 5′ mRNA, we find that m^6^A is distributed predominantly near stop codons. Interestingly, 85 % of the modified transcripts show high m^6^A methylation extent compared to their transcript level. The 290 highly methylated transcripts are mainly associated with transporters, stress responses, redox, regulation factors, and some non-coding RNAs. On average, the proportion of transcripts showing differential methylation between two plant organs is higher than that showing differential transcript levels. The transcripts with extensively higher m^6^A methylation in an organ are associated with the unique biological processes of this organ, suggesting that m^6^A may be another important contributor to organ differentiation in *Arabidopsis*. Highly expressed genes are relatively less methylated and *vice versa*, and different RNAs have distinct m^6^A patterns, which hint at mRNA fate. Intriguingly, most of the transposable element transcripts maintained a fragmented form with a relatively low transcript level and high m^6^A methylation in the cells.

**Conclusions:**

This is the first study to comprehensively analyze m^6^A patterns in a variety of RNAs, the relationship between transcript level and m^6^A methylation extent, and differential m^6^A patterns across organs in *Arabidopsis*.

**Electronic supplementary material:**

The online version of this article (doi:10.1186/s13059-015-0839-2) contains supplementary material, which is available to authorized users.

## Background

Over 100 types of chemical modifications have been discovered in RNAs from all of the living species [1, 2]. The most diverse modifications were present in ribosomal RNA (rRNA) and transfer RNA (tRNA). Chemical modifications are also prevalent in messenger RNA (mRNA) and other non-coding RNA (ncRNA) in eukaryotes [1, 2]. Among those, the most important is modified by *N*^6^-methyladenosine (m^6^A) [2–4]. m^6^A has been found ubiquitously distributed in rRNA, tRNA, mRNA, and some snRNA of eukaryotes, such as yeast [5], mammals [4, 6], insects [2], and plants [7]. Recently, transcriptome-wide analyses showed that one-third of the transcribed genes (mRNA) were modified by m^6^A in human and mouse [4, 6, 8]. The m^6^A enriched sites were found near stop codons, in 3′UTRs and mRNA segments derived from large exons [4, 6, 8]. These studies also showed that this modification was highly conserved in eukaryotes [2, 6], suggesting that a delicate regulatory mechanism may be responsible for this selective modification, and provided clues of the important metabolisms that this modification involved in or was responsible for, for example, RNA splicing [6, 8], RNA export [4], and RNA stability [4, 6].

The availability of antibody that specifically binds the m^6^A modified sites and efficiently enriches RNAs containing m^6^A modification facilitates the transcriptome-wide analysis of the patterns of this RNA modification through the biotechnologies of RNA sequencing (RNA-seq), RNA immunoprecipitation (RIP), and m^6^A-seq [4, 6, 8]. RIP was primarily used to analyze RNA-protein interaction [9]. However, the aim of the RIP experiment for the m^6^A-seq study was to pull down the RNA of interest containing m^6^A modification through application of m^6^A antibody to the randomly fragmented RNA pool. m^6^A-seq is a recently reported technology integrating the powers of both RIP and high-throughput RNA sequencing for transcriptome-wide analysis of m^6^A methylation patterns in eukaryotes [6, 10].

Transcriptome-wide analysis of m^6^A in mammals and plants provided insights into topological patterns and facilitated discovery of some functions of this RNA modification [4, 6, 10–13]. However, the differential m^6^A methylation among plant organs, for example, leaves, flowers, and roots, has not been well characterized. In this study, we significantly improved biotechnologies for RNA isolation and RIP, thus deep and high quality m^6^A-seq and massively m^6^A-mapped datasets in *Arabidopsis* are now available. This study aimed to: (1) comprehensively and transcriptome-wide characterize the m^6^A distributing patterns in numerous types of RNAs in *Arabidopsis*; (2) analyze the relationship between the transcript level and the m^6^A modification extent in the *Arabidopsis* transcriptome; (3) characterize differential patterns of the m^6^A methylation among leaves, flowers, and roots; and (4) discuss new functions of m^6^A modification in the transcripts extensively modified by m^6^A from the clues of the potential biological functions in these transcripts. This is the first study to comprehensively analyze m^6^A differential patterns across organs in plants. This study opens up a new avenue to greatly understand the transcriptome-wide patterns of m^6^A modification in different RNAs, relationship between m^6^A methylation extent and gene transcript level, and m^6^A differential patterns across organs in plants.

## Results

### Quality and depth of the RNA sequencing in this study

Commercial m^6^A antibody has proved to specifically bind to m^6^A RNA and has been successfully used for the m^6^A RNA immunoprecipitation experiments in the previous studies [4, 6, 10–13]. In this study, we collected samples from three organs of *Arabidopsis*: leaves, flowers, and roots; and performed m^6^A-seq, mRNA-seq, and input RNA-seq (total fragmented RNA without RIP experiments for sequencing and as the control for m^6^A-seq) with two replicates for each sample (Additional file 1). A total of 90 to 156 million reads were generated for each m^6^A-seq sample; 48 to 92 million reads for each mRNA-seq sample and 25 to 53 million reads for each input RNA-seq sample (Additional file 1). The proportion of the cleanly mapped reads and transcripts in m^6^A-seq were around 65–70 % (Additional file 1). Compared to m^6^A-seq data in the mammalian (11 to 24 million reads for each sample) [6] and in the rice (23 to 47 million reads for each sample) [12], the depth of the m^6^A-seq in this study (Additional file 1) was greatly high. The HPLC-MS/MS results indicated that RIP efficiency for m^6^A enrichment was high in this study (Additional file 2), and the non-specific immunoprecipitation rate was extensively low (lower than 1 %) in this study (Additional file 2), suggesting that the experimental error caused by non-precipitation was also low in the m^6^A-seq in this study.

### General features and extent of m^6^A methylation in *Arabidopsis*

**Fig. 1:**
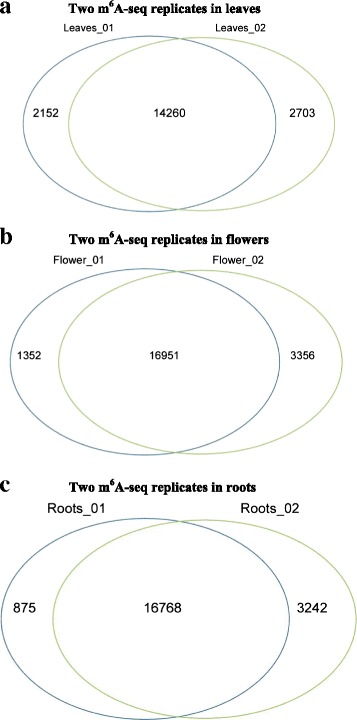
Number of overlapped m^6^A transcripts in the two m^6^A-seq replicates of three organs of leaves (**a**), flowers (**b**), and roots (**c**) in *Arabidposis*. The analysis indicated that over 83 % of m^6^A transcripts were overlapped in the two m^6^A-seq replicates

We identified that 16,688 to 19,305 transcripts were modified by m^6^A in the three *Arabidopsis* organs. For all three organs, at least 83 % agreement was found between two m^6^A-seq replicates in this study (Fig. 1). This agreement proportion between replicates was the highest compared to the previous reports [6, 13]. We found that 70.6 %, 73.7 %, and 76.7 % of the transcribed genes (transcripts) were chemically modified by m^6^A in the leaves, flowers and roots of *Arabidopsis*, respectively (Additional file 3). This estimation was greatly higher than previous reports (over one-third) in human, mouse [6] and (approximately 50 %) plant [13]. The estimation differences may be due to the different criteria used for calling of the ‘m^6^A modified transcripts’ in m^6^A-seq [6, 13]. In the previous studies, input data were used as the control for calling the m^6^A modified transcripts due to relatively high non-specific immuno-precipitation rate in their experiments. This could result in underestimation of both total m^6^A peaks and proportion of the m^6^A transcripts in the transcriptome [6, 13]. However, all the mapped reads after removal of PCR duplicates in the m^6^A-seq were counted and considered to be derived from RNA fragments containing m^6^A modification in this study. And thus the transcripts with m^6^A mapped reads were considered as the modified transcripts due to very low non-specific immuno-precipitation rate (lower than 1 %) in this study (Additional file 2). But our estimation may better reflect the m^6^A modification extent in the cells (see details in the Discussion section of this paper).

**Fig. 2:**
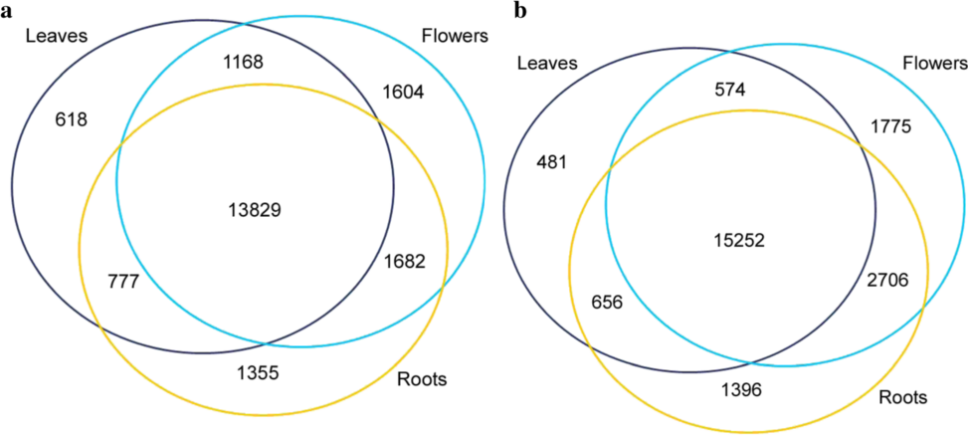
Number of the overlapped transcripts in the two (**a**, **b**) m^6^A-seq replicates

On average of two replicates, over 80 % of the m^6^A modified transcripts were common among the leaves, flowers, and roots (Fig. 2). On average, around 32,300 m^6^A sites from the leaves, approximately 43,400 sites from the flowers, and approximately 48,100 from the roots were successfully mapped to the *Arabidopsis* genome with an estimation of approximately 2.0 to 2.6 m^6^A sites per m^6^A transcript and approximately 1.4 to 2.0 m^6^A sites per transcript in the whole transcriptome (Additional file 4). This estimation was comparable to that in mammals (approximately 1.5 m^6^A sites per transcript) [6], but higher than the estimated in the plant (0.7 to 1.0 m^6^A site per transcript) [13]. However our observation was closer to the earlier reports, for example, approximately 3-5 m^6^A sites per transcript [1, 2]. The ratio of m^6^A/A in the m^6^A modified transcripts was in the range of 0.44 % to 0.61 % in the three organs, and this ratio was in a range of 0.35 % to 0.50 % in the whole transcriptome of three organs in *Arabidopsis* (Additional file 5). This ratio estimation is also comparable to the recently reported in the plant [13].

### m^6^A topological patterns in *Arabidopsis*

**Fig. 3:**
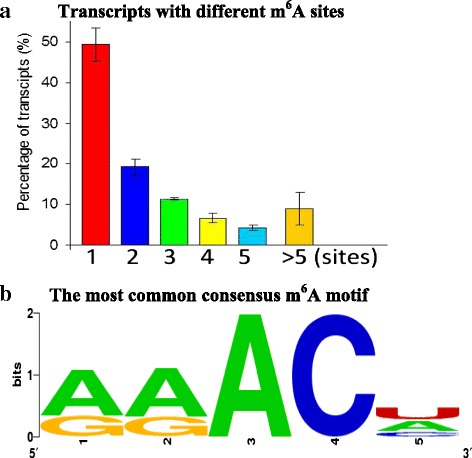
The general m^6^A patterns in *Arabidopsis*. **a** Proportion of the modified transcripts containing different m^6^A sites. **b** Sequence logo representing the most common consensus motif (RRm^6^ACH) in the m^6^A peaks in *Arabidopsis*

Over two-third of the methylated transcripts exhibited one or two m^6^A sites (Fig. 3a, details in Additional file 6). And over 17 % contained four or more sites (Fig. 3a, details in Additional file 6), which was much higher than previously reported (only 5.5 %) in human [6].

The consensus sequence of m^6^A modification has been identified as ‘RRm^6^ACH’, where R is A/G and H is A/C/U [4, 6, 14]. Our data showed that over 75 % of the RIP fragments in the m^6^A-seq contained the consensus sequence RRm^6^ACH in *Arabidopsis* (Fig. 3b). The most two frequent motifs were AAm^6^ACU (19.3 %) and AAm^6^ACA (19.0 %) (Fig. 3b). This observation is consistent with the recent reports in plants [12, 13].

**Fig. 4:**
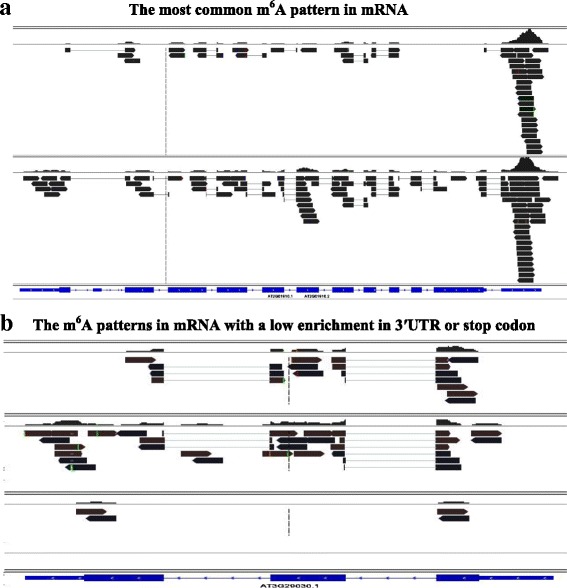
Schematic screen shots of two typical types of m^6^A topologies in mRNA in *Arabidopsis*. **a** Type 1 (representative gene, ‘AT2G01910’; trace files of two organs (flowers and roots) were presented), one or two predominant peaks in 3′UTR or at stop codon with several much lower signals in the codon regions. The peaks in 3′UTR or at stop codon were two to tens of folds of the signals in the codon regions. Most of the messenger RNA (over 70 %) presented this type of m^6^A topology. **b** Type 2 (representative gene, ‘At3g29030’; trace files of three organs, leaves (the upper), flowers (in the middle), and roots (the lower) were presented). Several m^6^A sites distributing in the transcripts with low m^6^A signals (in the middle and lower parts of the figure, representative of flowers and roots) or without peaks in 3′UTR or at stop codon (in the upper part, representative of leaves)

**Fig. 5:**
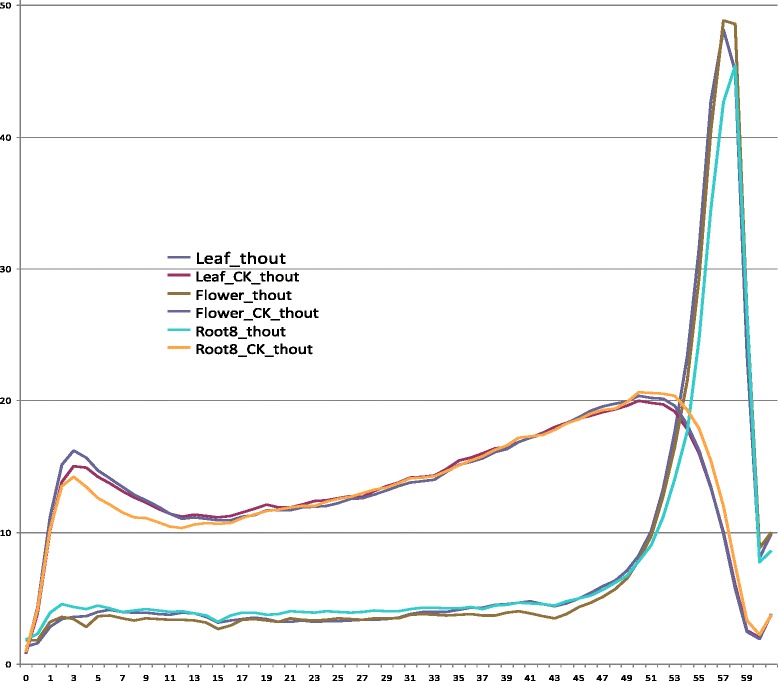
The overall m^6^A distributing pattern from 5′ (left) to 3′ (right) in the m^6^A-seq datasets and the overall transcriptional pattern from 5′ (left) to 3′ (right) in the mRNA-seq datasets in the three organs of *Arabidopsis*. The patterns with the caption of ‘CK’ in the image was deduced from the mRNA-seq datasets. The other threes were from the m^6^A-seq dataset. The number ‘0’ on the left refers to TSS. The numbers from 49 to 59 represents stop codon or the proximate 3′UTR. As shown in this figure, only one dominant peak of m^6^A enrichment was detected around 3′UTR or stop codon in the overall dataset of the *Arabidopsis* transcriptome in this study

The m^6^A topology in mRNA was categorized into two types according to the m^6^A patterns distributing in the whole transcript. One type was characterized by dominant m^6^A enrichment observed at stop codon or 3′UTR. And 73.0 % to 76.3 % of the mRNA in three organs was modified by this type of the m^6^A pattern (Fig. 4a, Additional file 7). Thus, most of the methylated mRNA was characterized by this typical m^6^A topology in *Arabidopsis* (Type 1, Fig. 4a): one or two high peaks at stop codon or at 3′UTR with extremely low m^6^A signals observed in the coding regions. In most cases, the peak height in 3′UTR or at stop codon was two to a dozen of folds of the signal heights in the coding regions (Fig. 4a, Fig. 5). This dominant m^6^A enrichment was not found in the remaining mRNA (Type 2, Fig. 4b). The overall m^6^A signals were also relatively low in Type 2 (Fig. 4b). Transcriptome-wide analysis showed that the overall m^6^A patterns distributing within genes were highly close with each other among three organs (Fig. 5). Statistic analysis indicated that the normalized read depth representing the overall m^6^A patterns had non-significant differences among three organs (*P* = 0.716, Additional file 8), suggesting that recognition of motif for m^6^A methylation was extensively conserved among plant organs.

**Fig. 6:**
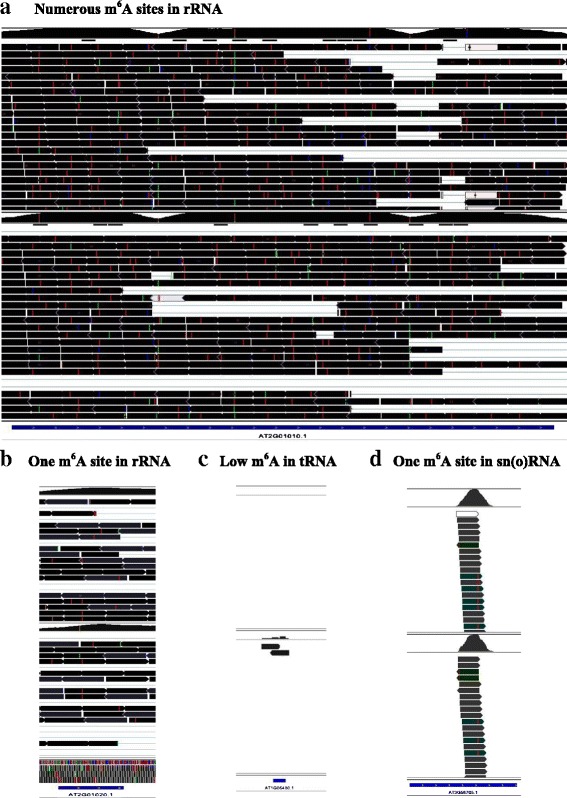
Schematic screen shots of the m^6^A patterns in rRNA, tRNA and sn(o)RNA. **a** Numerous m^6^A sites distributed in a rRNA transcript (reprehensive ‘AT2g01010’). **b** A single m^6^A in a rRNA transcript (representative ‘AT2g01020’). **c** Low m^6^A methylation in most of tRNA (approximately 90 %, reprehensive ‘AT1g06480’). **d** sn(o) RNA was highly methylated by a single m^6^A site (representative ‘AT3G56705’)

Two types of m^6^A patterns were observed in rRNAs: one was modified by one m^6^A site, and the other was methylated by several m^6^A sites (Fig. 6a and b). The m^6^A topology in tRNAs was also categorized into two major types: approximately 10 % of tRNAs were slightly modified by m^6^A (Fig. 6c) and m^6^A methylation was not observed in the remaining tRNAs. Both snRNA and snoRNA were highly methylated by m^6^A, but only a single m^6^A site was found in these two types of RNAs (Fig. 6d). Therefore, different types of RNAs were endowed with distinct m^6^A topologies.

### m^6^A methylation extent versus gene transcript level

To compare m^6^A methylation extent in m^6^A-seq with gene transcript level (TL) in mRNA-seq in three organs of *Arabidopsis*, we categorized the m^6^A methylation extent into three groupings based on the comparison of modified Fragments Per Kilobase of Transcript Per Million Fragments Mapped (MFPKM) of the transcript in the m^6^A-seq with the FPKM of the counterpart in the mRNA-seq using *χ*^2^ test (to test whether ratio of MFPKM to FPKM in each transcript fits 1:1 in each organ, *P* <0.05) (Table 1). The ratio of MFPKM to FPKM is higher (or lower) than 1 by *χ*^2^ test (*P* <0.05), representing that the m^6^A methylation extent in m^6^A-seq was relatively high (or low) compared to the transcript level in mRNA-seq. When the ratio of MFPKM to FPKM in each transcript fits 1:1 (*P* <0.05), the m^6^A methylation extent was considered relatively ‘equivalent’ to the transcript level. This comparison was applied to estimate the m^6^A methylation extent of a transcript compared to its transcript level. All the m^6^A methylated transcripts were used for this estimation and the transcripts without m^6^A methylation detected were excluded for this comparison.

**Table 1:** Three groupings of the m^6^A methlylation extent compared to the transcript level in three organs of the *Arabidopsis* m^6^A transcriptome

Replicates	Plant organs	High		Low		Equivalent	
		Transcripts (n)	Proportion (%)	Transcripts (n)	Proportion (%)	Transcripts (n)	Proportion (%)
Replicate 1	Leaves	13,711	83.5	606	3.7	2,094	12.8
	Flowers	14,807	80.8	762	4.2	2,760	15.0
	Roots	14,275	80.9	413	2.3	2,955	16.7
	Average		81.7		3.4		14.8
Replicate 2	Leaves	16,067	94.7	98	0.6	798	4.7
	Flowers	18,764	92.4	100	0.5	1,443	7.1
	Roots	17,188	85.9	485	2.4	2,337	11.7
	Average		91.0		1.2		7.8

‘High’, ‘Low’, and ‘Equivalent’ were categorized by comparison of the m^6^A-seq depth (MFPKM, the methlylation extent of m^6^A) of each transcript with that in the mRNA-seq (FPKM, the transcript level). ‘High’ or ‘Low’ referred to as a relatively high or low m^6^A methlylation extent compared with its transcript level based on *χ* ^2^ test (*P* <0.05); ‘Equivalent’, suggested that the m^6^A methlylation depth was relatively ‘equivalent’ to the transcript level (ratio of MFPKM to FPKM fits 1:1) based on *χ* ^2^ test (*P* <0.05)

Interestingly, 83.4 % to 89.1 % of the methylated transcripts showed a high m^6^A modification level, while <4 % of the m^6^A modified transcripts had a low modification extent versus their transcript level in mRNA-seq in the three organs (Table 1). Thus, most of the m^6^A modified transcripts showed a relatively high m^6^A modification extent versus their gene transcript level in the three organs of *Arabidopsis*.

To further analyze relationship between the m^6^A methylation extent in m^6^A-seq and the transcript level in mRNA-seq in three organs of *Arabidopsis*, the transcript level was categorized into three groupings: high, moderate and low. Each category contained one-third of the m^6^A modified transcripts from the highest to the lowest FPKM in mRNA-seq. The comparison of ratio of the average MFPKM in m^6^A-seq to the average FPKM in mRNA-seq between three groupings using *t*-test (Table 2) showed that most of the highly expressed transcripts were relatively less modified by m^6^A, and most transcripts with a low expression level were more likely modified by m^6^A (*P* <0.005). The moderately expressed transcripts tended to be moderately methylated in *Arabidopsis* (*P* <0.005). This observation somewhat differed from the report in human and mouse: the moderately expressed transcripts were more likely to be methylated, and those expressed at the two extremes were less methylated by m^6^A [6]. Both of our two groups used the similar methods to analyze the relationship between the m^6^A methylation extent and the gene expression level [6]. The reasons causing the differences may not be due to different methodologies or different biological species because a number of studies have proved that m^6^A methylation is highly conserved among eukaryotes [2, 6, 8, 12]. However, our observation may authentically reflect m^6^A methylation in the cells: the genes with a lower transcript level may require a relatively higher m^6^A modification extent to maintain RNA stability in the cells [12, 13] and *vice versa*.

**Table 2:** Relationship between the m^6^A methlylation extent and the transcript level in three organs of the *Arabidopsis* m^6^A transcriptome

Replicates	Plant organs	High			Moderate			Low		
		MFPKM	FPKM	Ratio	MFPKM	FPKM	Ratio	MFPKM	FPKM	Ratio
Replicate 1	Leaves	232.23	75.65	3.07	146.87	29.17	5.03	76.96	7.66	10.05
	Flowers	206.13	65.15	3.16	137.14	28.56	4.80	68.52	8.68	7.89
	Roots	156.63	62.28	2.51	111.23	28.44	3.91	56.22	7.35	7.65
Replicate 2	Leaves	221.14	88.55	2.50	176.68	17.94	9.84	99.06	3.75	26.42
	Flowers	152.77	80.11	1.91	120.33	15.29	7.87	69.34	3.49	19.87
	Roots	98.93	83.58	1.18	65.81	11.74	5.61	45.53	2.22	20.49

‘High’, ‘Moderate’, and ‘Low’ refer to three groupings of the transcript levels from the highest to the lowest FPKM in mRNA-seq. Each grouping included one-third numbers of the m^6^A modified transcripts. *t*-test on ratio of the average MFPKM in m^6^A-seq to the average FPKM in mRNA-seq in each grouping showed significantly different (*P* <0.005) ratios between three groupings

**Fig. 7:**
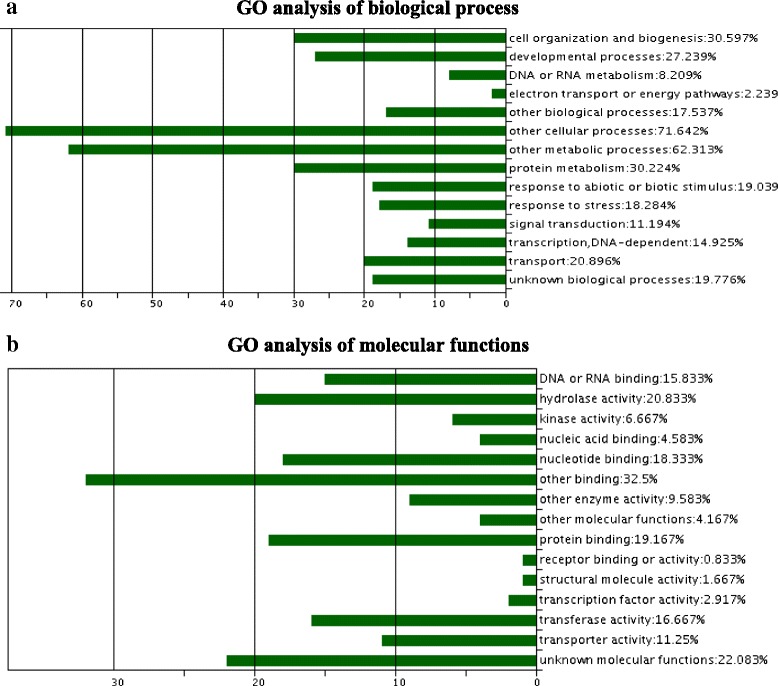
GO analysis of the biological process (**a**) and molecular functions (**b**) for the 290 transcripts extensively methylated by m^6^A in the *Arabidopsis* transcriptome. Proportions in the figures indicated the level of the hits for each classification in the blast. As the majority of mRNA was methylated by m^6^A, methylation occurs on a functionally distinct subset of transcripts. However, most of the gene transcripts extensively methylated by m^6^A were related to the proteins that they were a direct transporter for energy molecules, for example, ATP or GTP, or accomplishment of their biological functions was dependent on these energy chaperones

We found that approximately 5.5 % of the m^6^A modified transcripts were extensively methylated by m^6^A in each organ (with fold change (ratio of MFPKM in the m^6^A-seq to FPKM in the mRNA-seq) ≥10, false discovery rate (FDR) <10^−12^, and read number per transcript ≥30). And 290 (2.1 %) extensively m^6^A methylated transcripts were found common in all of the three organs of leaves, flowers, and roots in *Arabidopsis*. These transcripts extensively modified by m^6^A were mainly associated with transporter, defenses or stress response, redox, nucleic acid binding, signal transduction, regulation of transcription, DNA/RNA/protein modification, cell proliferation or cycle, transposable element gene, pseudogenes, and snRNA (Table 3) [15–63]. Gene ontology (GO) analysis showed that the major molecular functions in these transcripts were responsible for molecule binding, transferase, hydrolase, stress responses, transporter, and kinase activity (Fig. 7).

**Table 3:** Potential functions in the 290 transcripts presenting extensively high methylation in all of the three organs of leaves, flowers and roots in *Arabidopsis*

Potential functions^a^	Gene ID	References^b^
Transporter	AT1G23900, AT1G06470, AT1G60070, AT1G79610, AT2G07671, AT2G07687, AT2G07698, AT2G07741, AT2G41700, AT3G08650, AT3G08960, AT3G17430, AT3G20560, AT3G20920, AT3G46830, AT4G00630, AT4G00800, AT4G38920, AT4G39850, AT5G27970, AT5G36940, AT5G53530, AT5G01990, AT3G55320, AT4G13750, AT1G16820, AT1G77140, AT5G05570, AT5G07770, AT2G21340, AT2G27460, AT1G74720, AT1G47550, AT5G66380, AT5G47490, AT5G08470, AT3G03720, AT5G61310, AT5G62600, AT5G11980, AT1G56290, AT2G20840, AT2G15240, AT4G39420, AT5G07980,	[15–21]
Defense or stress response	AT1G63770, AT1G64790, AT1G67090, AT1G80030, AT2G05580, AT2G27380, AT2G35510, AT2G42560, AT3G20290, AT3G22640, AT3G49600, AT4G01210, AT4G04920, AT4G08230, AT4G25520, AT4G33650, AT5G10450, AT5G11530, AT5G14030, AT5G14790, AT5G34850, AT5G35620, AT5G43460, AT5G63110, AT3G46920, AT1G10522, AT2G46240, AT1G58220, AT1G57870, AT1G01260, AT1G67890, AT4G31390, AT3G54610, AT1G74720, AT1G80010, AT1G31835, AT1G50730, AT4G05631	[18, 22–33]
Redox process	AT1G50430, AT1G67140, AT1G76150, AT2G27110, AT1G80560, AT2G07687, AT2G07727, AT2G38020, AT3G20560, AT2G48060, AT4G08280, AT4G23420, AT4G39850, AT5G42790, AT5G65750, AT4G01860, AT3G08950, AT3G01380, AT4G36080, AT2G43420, AT4G16310, AT5G21060, AT1G56000, AT4G17150, AT5G08470, AT4G16070, AT4G30993, AT3G20560	[18, 34–36]
Signal transduction	AT1G03060, AT1G43130, AT1G48090, AT1G51690, AT1G58250, AT3G46830, AT3G49600, AT4G38200, AT5G06260, AT5G28900, AT5G35180, AT5G49470, AT5G39760, AT2G15240, AT3G55850, AT1G13180, AT1G10522, AT1G67890, AT5G07770, AT5G06350, AT4G02970, AT1G58230, AT2G46700, AT3G18040, AT5G07770, AT1G74720	[19, 28, 33, 37–39]
Nucleic acid binding, DNA repair, DNA/RNA synthesis	AT1G02990, AT1G08840, AT1G12930, AT1G50840, AT2G20000, AT2G32000, AT1G17580, AT3G54280, AT1G20920, AT1G58060, AT2G19520, AT3G23780, AT3G48190, AT3G61240, AT4G09680, AT4G25880, AT5G05560, AT5G16630, AT5G22010, AT2G03070, AT3G23780, AT3G53500, AT1G07705, AT4G00060, AT4G16280, AT4G32200, AT1G33390, AT3G54460	[18, 40–43]
Regulation of transcription	AT1G07470, AT2G20330, AT2G35110, AT3G53500, AT4G04920, AT5G42770, AT5G49470, AT5G63260, AT1G07705, AT1G53541, AT5G39760, AT1G17450, AT1G10522, AT5G13240, AT1G58220, AT1G01260, AT5G08230, AT2G48110, AT1G33390, AT5G08550, AT2G36960, AT3G61740, AT3G10070, AT5G49430	[18, 30, 44]
DNA methylaytion, demethylation, and gene silencing	AT1G08060, AT1G54490, AT2G06210, AT3G01460, AT4G16280, AT3G07610, AT5G05570	[18, 45, 46]
Cell proliferation, circadian rhythm, or differentiation	AT1G17110, AT1G17580, AT1G22620, AT1G22770, AT1G67490, AT2G25730, AT2G26890, AT2G35110, AT3G07160, AT3G49600, AT5G06265, AT5G11030, AT5G12980, AT5G24740, AT5G40740, AT5G42770, AT5G51290, AT2G19390, AT1G55540, AT3G15120, AT5G10340, AT5G48120, AT1G77460, AT4G13750, AT3G27670, AT4G32200, AT4G04970, AT3G19630, AT4G18600, AT1G11060, AT4G02070, AT1G67140	[18, 21, 40, 47–51]
Protein phosphorylation or histone acetylation	AT1G13320, AT1G16710, AT1G49340, AT5G04510, AT5G18525, AT5G49470, AT3G46920, AT1G31860, AT1G57870, AT4G31390, AT2G46700, AT3G18040	[18, 52, 53]
Protein post translational process, for example, folding, ubiquitination	AT1G62330, AT3G06440, AT3G18520, AT3G56120, AT3G59410, AT4G33650, AT5G05920, AT5G06260, AT5G11530, AT5G51660, AT5G63110, AT1G79940, AT1G80030, AT1G73950, AT3G60350, AT5G07910, AT3G54610, AT1G80010, AT3G46220, AT3G20560, AT1G56290	[18, 54, 55]
RNA post-transcriptional processing	AT1G24050, AT1G24706, AT1G31870, AT1G32500, AT1G64572, AT3G11540, AT1G35470, AT1G73720, AT3G11960, AT3G19670,	[18, 56–59]
	AT3G47890, AT3G53500, AT3G53500, AT5G51660, AT3G56825, AT3G57570, AT3G19515, AT3G19630, AT3G13290, AT5G10370, AT4G02970, AT5G62600, AT3G55220, AT3G10070	
Proteolysis or protein synthesis	AT1G67120, AT1G67550, AT2G40930, AT4G26510, AT5G35620, AT5G58200, AT5G23110, AT1G28350, AT5G49030, AT5G27700, AT3G47060, AT2G07715, AT2G24640, AT2G25740	[18]
Protein located in mitochondria or chloroplast	AT1G09980, AT1G58350, AT1G68160, AT2G01008, AT2G07671, AT2G07708, AT2G07687, AT2G07727, AT2G11910, AT2G31141, AT2G33980, AT2G35750, AT2G07698, AT3G12590, AT3G41762, AT3G50380, AT4G00585, AT4G02770, AT4G31350, AT4G38120, AT4G39690, AT5G08060, AT5G15320, AT5G15750, AT5G26850, AT5G59613, AT1G07705, AT3G58010, AT3G63052, AT1G30910, AT3G08950, AT3G47060, AT2G07715, AT1G10522, AT3G06310, AT1G31860, AT5G53740, AT1G49700, AT4G31390, AT2G21340, AT3G43540, AT2G25660, AT5G66380, AT1G45332, AT5G61310, AT5G15700, AT3G18040, AT3G56120, AT4G01210, AT4G00630, AT4G13730, AT4G38920, AT5G53530, AT1G63770	[17, 18, 28, 29, 60–63]
Transposable element gene	AT3G28945, AT4G06477, AT4G08114, AT4G08115, AT5G35935, AT3G42806, AT4G16870, AT4G08112	
Pseudogenes	AT2G07709, AT2G07711,AT2G07717, AT2G07733, AT2G07811, AT2G07747, AT2G35743	
sn (o) RNA or other ncRNA	AT1G15405, AT1G08115, AT3G56705, AT5G09585, AT5G61455,	
	AT3G55485, AT2G01020, AT1G16635, AT2G01010, AT2G43375, AT3G56825, AT2G46192, AT5G06165, AT1G61275, AT4G39363, AT3G41979, AT1G12013	
RNA post-transcriptional processing	AT1G24050, AT1G24706, AT1G31870, AT1G32500, AT1G64572, AT3G11540, AT1G35470, AT1G73720, AT3G11960, AT3G19670,	[18, 56–59]
	AT3G47890, AT3G53500, AT3G53500, AT5G51660, AT3G56825, AT3G57570, AT3G19515, AT3G19630, AT3G13290, AT5G10370, AT4G02970, AT5G62600, AT3G55220, AT3G10070	

^a^Suggests the function of RNA itself, for example, rRNA, or the functions in its expressed proteins

^b^The functions of many transcripts were inferred by gene ontology (GO) analysis using the online tool in TAIR (http://www.arabidopsis.org/) and some functions were inferred from the recent publications

### Differential m^6^A methylation among leaves, flowers, and roots

High m^6^A sequencing depth (reads) of a transcript in the m^6^A-seq dataset may not suggest that this transcript was highly methylated by m^6^A in the cells because the gene transcript level of the counterpart may also be relatively high in the mRNA-seq dataset. To minimize effect of gene transcript level on estimation of m^6^A methylation extent of the transcripts and to more precisely evaluate differential m^6^A methylation among organs, we applied an algorithm ‘NFPKM’ to each transcript to estimate differential m^6^A methylation among three organs (see details in the Methods section of this paper). Two fold change and chi-square were applied for estimation of differential m^6^A methylation between organs using our algorithm. Accordingly, these two criteria were also used to estimate differential gene transcripts in mRNA-seq and to have a parallel comparison with the results of differential m^6^A methylation among organs in this study.

On average, 26.6 % of the transcripts presented differential in mRNA-seq (fold change of FPKM between two organs >2 or <0.5, and FDR <0.05), while 33.5 % showed differential methylation between two organs (fold change of NFPKM between two organs >2 or <0.5, and FDR <0.05) (Table 4). A paired analysis indicated that ratio of transcripts showing differential m^6^A methylation was significantly higher than that showing the differential gene transcripts in the three *Arabidopsis* organs (*P* <0.00035). The comparison also showed that the leaves had the highest extent of m^6^A methylation among three organs followed by the flowers. And the roots were less likely methylated among three organs (Table 4).

**Table 4:** The gene transcripts presenting differential transcript level and differential m^6^A methylation across three organs in *Arabidopsis* (fold change >2 or <0.5, FDR <0.05)

Replicates and differential transcripts			Leaves vs. Flowers		Leaves vs. Roots		Flowers vs. Roots	
			Hi-leaves	Hi-flowers	Hi-leaves	Hi-leaves	Hi-flowers	Hi-root
Differential transcript level	Replicate 1	Transcripts (n)	893	1,826	1,706	1,869	2,070	1,148
		Proportion (%)	6.5	13.2	12.3	13.5	15.0	8.3
		Total (%)	19.7		25.8		23.3	
	Replicate 2	Transcripts (n)	1,728	2,671	2,724	2,031	3,068	1,626
		Proportion (%)	11.3	17.5	17.9	13.3	20.1	10.7
		Total (%)	28.8		31.2		30.8	
Differential m^6^A methylation	Replicate 1	Transcripts (n)	2,273	1,601	2,857	1,537	2,451	1,649
		Proportion (%)	16.4	11.6	20.7	11.1	17.7	11.9
		Total (%)	28.1		31.2		29.6	
	Replicate 2	Transcripts (n)	4,004	1,268	4,819	1,869	4,576	1,062
		Proportion (%)	26.3	8.3	31.6	12.3	30.0	7.0
		Total (%)	34.6		43.8		37.0	

Eleven genes were randomly chosen for validation of our analysis above (Additional file 9). As products of qRT-PCR cover a short span in the transcriptome (50 to 150 bp) [13], two flanks of the amplicon containing one m^6^A peak in IGV program and showing differential m^6^A methylation were chosen to design primers (Additional file 9). The correlation coefficient between the qRT-PCR relative abundance results and the RIP-seq expected abundances was significant (*r* = 0.8632 (*r* between 0.7738-0.9526 for 95 % % CI), *n* = 33 genes, and *P* <10^−4^), indicating that our qRT-PCR data were consistent with the data estimated by m^6^A-seq and mRNA-seq using the IGV program (Additional file 10).

**Fig. 8:**
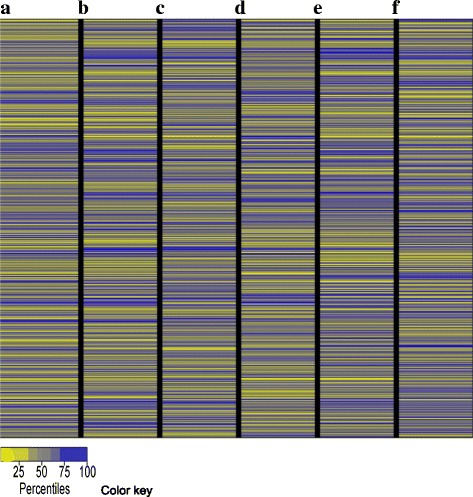
Heatmap by the gene alphabetic order presenting the overall differential patterns of both gene transcript and m^6^A methylation level in the transcripts in *Arabidopsis*. **a** Overview of the differential patterns of m^6^A methylation between leaves and flowers. **b** Overview of the differential patterns of m^6^A methylation between leaves and roots. **c** Overview of the differential patterns of m^6^A methylation between flowers and roots. **d** Overview of the differential patterns of gene transcript level between leaves and flowers. **e** Overview of the differential patterns of gene transcript level between leaves and roots. **f** Overview of the differential patterns of gene transcript level between flowers and roots. The patterns in all of the six comparisons (**a** to **f**) above were based on the alphabetic order of the gene ID representing Chromosomes 1 to 5 (up to down)

The heatmap representing the overall patterns of both differential transcript level and m^6^A methylation extent revealed completely distinct patterns: (1) between transcript level and m^6^A methylation; (2) among three organs; (3) among different genes; (4) among different regions on the same or different chromosomes; and (5) among five chromosomes (Fig. 8). This suggested that both differential gene transcripts and differential m^6^A methylation were highly heterogeneous in the *Arabidopsis* transcriptome and that regulation of the gene transcript level and extent of RNA m^6^A methylation may be relatively independent events.

**Fig. 9:**
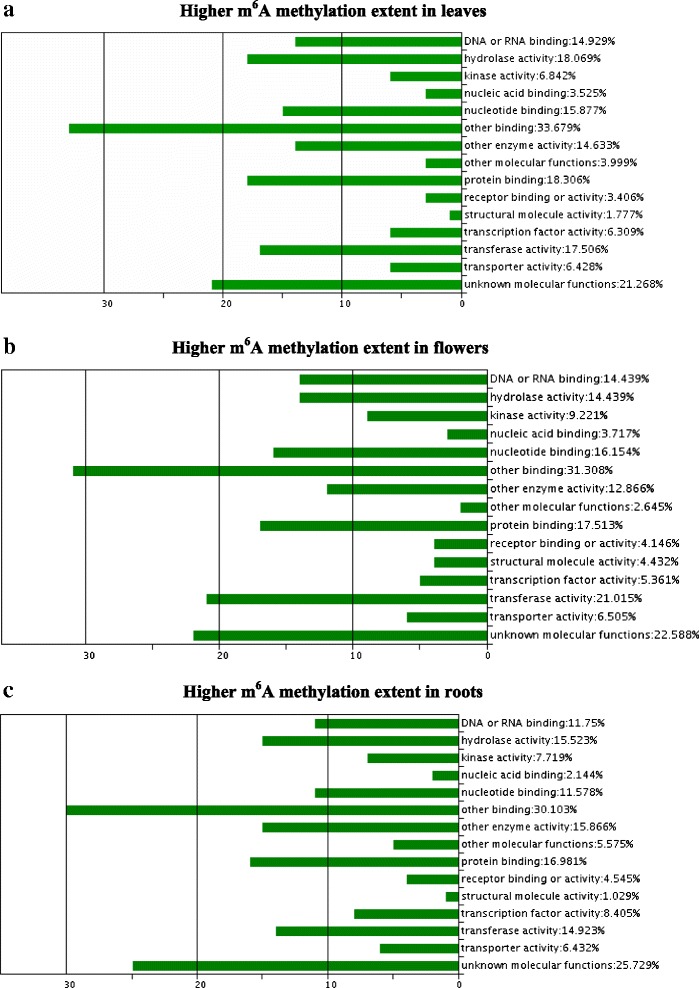
GO analysis of molecular functions of transcripts presenting a higher extent of m^6^A methylation in leaves (**a**), flowers (**b**), or roots (**c**) than other two organs in *Arabidopsis*

Analysis of common elements between two replicate datasets showed that 2,628 (18.1 %) m^6^A modified transcripts in leaves, 1,920 (13.5 %) in flowers, and 1,166 (8.0 %) in roots showed a higher extent of methylation than the other two organs (fold change of NFPKM between two organs >2 or <0.5, FDR <0.05). Based on GO analysis, the major molecular functions in these transcripts were relevant to binding activity, transferase, hydrolase, kinase, transporter, and transcription factor (Fig. 9). Based on KEGG pathway analysis, certain transcripts presenting higher methylation in leaves than the other two organs were related to pathways of photosynthesis, carbohydrate, and nitrogen metabolism (Table 5); transcripts with higher methylation in flowers were concerning metabolic pathways of RNA degradation, DNA replication, and protein synthesis metabolisms (Table 5); transcripts presenting higher methylation in roots involved in biosynthesis of alkaloids, and carbonate metabolism (Table 5) (fold change of NFPKM between two organs >2 or <0.5, FDR <0.005). We found that 43 transcripts in leaves, 41 in flowers, and 23 in roots showed an extensively higher methylation level than the other two organs (fold change of NFPKM between two organs ≥10 or <0.1, FDR <10^−10^, and read number per transcript ≥20). The transcripts extensively methylated in leaves were mainly expressed for proteins located in mitochondria or chloroplast, photosynthesis, regulation of transcription, or stress response (Table 6) [64–71]; those in flowers (Table 7) were differentially or specifically expressed in the reproductive organs during flowering, or for cell proliferation, circadian rhythm, protein metabolism, transporter, or defense response [72–88]; and those in roots were mainly expressed for defense or stress response, transporters, redox process, or signaling transduction (Table 8) [89–100]. Therefore, the transcripts representing extensively higher m^6^A methylation in an organ revealed an intriguing phenomenon that functions of these transcripts were required for or highly related with unique biological roles of this organ (Tables 6, 7, and 8).

**Table 5:** KEGG pathways of the transcripts representing a higher methylation extent in one organ than the other two organs in *Arabidopsis*

Organs	Pathways	Count	Hit (%)	*P* value	Genes
Higher in leaves	Photosynthesis	16	0.66	3.72E-11	AT3G47470, AT1G29920, AT1G29930, AT1G61520, AT3G54890, AT3G27690, AT1G15820, AT3G08940, AT5G54270, AT4G10340, AT2G05100, AT2G34430, AT2G34420, AT2G05070, AT3G61470, AT5G01530
	Photosynthesis	28	1.15	3.01E-08	AT1G30380, AT2G20260, AT1G44575, AT5G66570, AT4G03280, AT4G05180, AT4G04640, AT1G76100, AT1G31330, AT1G03130, AT4G32260, AT1G52230, AT4G12800, AT4G02770, AT1G06680, AT1G60950, AT1G79040, AT1G55670, AT1G20340, AT4G09650, AT5G66190, AT1G15700, AT5G64040, AT3G16140, AT1G67740, AT4G28750, AT1G32550, AT3G50820
	Carbon fixation in photosynthetic organisms	23	0.94	4.67E-05	AT1G70580, AT3G55800, AT2G21170, AT5G65690, AT3G12780, AT3G54050, AT2G01290, AT2G45290, AT2G19900, AT5G61410, AT3G60750, AT5G11520, AT5G38410, AT5G38420, AT3G26650, AT1G32060, AT1G67090, AT1G68750, AT3G47520, AT5G38430, AT4G38970, AT5G52920, AT3G04790
	Nitrogen metabolism	11	0.45	0.03	AT3G03910, AT3G01500, AT3G23490, AT5G35630, AT2G28210, AT1G77760, AT5G14740, AT1G70410, AT1G11860, AT3G47340, AT2G41560
	Glycosaminoglycan degradation	4	0.16	0.03	AT5G13690, AT1G05590, AT5G27730, AT1G65590
	Glyoxylate and dicarboxylate metabolism	9	0.37	0.03	AT3G21720, AT3G47520, AT5G38430, AT5G03860, AT5G38410, AT4G17360, AT3G14415, AT5G38420
Higher in flowers	RNA degradation	7	0.60	0.03	AT5G38890, AT1G03330, AT3G07750, AT1G65700, AT1G03360, AT1G80780, AT3G03710
	DNA replication and protein synthesis	19	1.63	0.05	AT2G34480, AT5G64650, AT1G56045, AT3G25520, AT1G80750, AT3G09500, AT3G04840, AT4G34620, AT3G28500, AT5G02610, AT4G31985, AT2G01250, AT2G19720, AT2G04390, AT1G61580, AT2G25210, AT1G07070, AT5G39850, AT1G78630
Higher in roots	Flavonoid biosynthesis	9	0.46	1.33E-05	AT5G07990, AT1G74550, AT4G34050, AT5G42800, AT2G30490, AT3G55120, AT5G08640, AT5G13930, AT4G22880
	Carbonate metabolism	13	0.66	0.003	AT3G55800, AT3G54050, AT4G37870, AT4G26520, AT2G19900, AT4G26530, AT5G38420, AT1G42970, AT5G09660, AT5G11670, AT5G38430, AT1G12900, AT4G38970
	Biosynthesis of phenylpropanoids	28	1.43	0.006	AT1G74550, AT1G18870, AT3G54050, AT5G28237, AT4G26530, AT1G42970, AT2G37040, AT5G50950, AT4G34230, AT1G51680, AT5G47000, AT3G17070, AT2G37130, AT4G26520, AT5G51890, AT1G15950, AT5G07990, AT5G09660, AT3G21240, AT4G34050, AT1G12900, AT4G38970, AT2G30490, AT3G53260, AT3G55120, AT5G08640, AT5G13930, AT4G22880
	alpha-Linolenic acid metabolism	7	0.36	0.009	AT4G15440, AT3G45140, AT2G35690, AT2G06050, AT1G55020, AT1G76680, AT3G25780
	Phenylpropanoid biosynthesis	14	0.72	0.02	AT1G74550, AT3G17070, AT2G37130, AT5G51890, AT1G15950, AT4G34050, AT3G21240, AT2G37040, AT2G30490, AT3G53260, AT4G34230, AT2G22990, AT1G51680, AT5G47000
	Tropane, piperidine and pyridine alkaloid biosynthesis	5	0.26	0.03	AT2G29320, AT2G37040, AT2G29360, AT2G29340, AT3G53260
	Stilbenoid, diarylheptanoid and gingerol biosynthesis	10	0.51	0.03	AT1G74550, AT4G37310, AT4G34050, AT3G26220, AT3G26200, AT2G30490, AT5G04660, AT3G53280, AT3G26280, AT3G26290

**Table 6:** Potential functions of the 43 transcripts presenting extensively higher m^6^A methylation in leaves than that in the other two organs in *Arabidopsis* (fold change >10 or <0.1, FDR <10^−10^)

Potential functions^a^	Gene ID	References^b^
Carbohydrate metabolism	AT1G33700, AT5G13000	[18]
Photosynthesis metabolism or photomorphogenesis	AT1G55670, AT2G05100, AT3G08940, AT3G21055, AT1G67740, AT1G03130, AT2G34420, AT1G15820, AT4G10340, AT1G61520, AT2G06520, AT3G27690, AT3G15190, AT1G14790	[64–66]
Defense or stress response	AT4G35770, AT5G66570, AT2G05520, AT5G22690, AT5G42900, AT2G26650, AT1G70060, AT1G64060, AT1G17750, AT3G07770	[18, 67, 68]
Oxidation-reduction process	AT1G55670	[69, 70]
Protein located in mitochondria or chloroplast	AT1G55670, AT2G05100, AT3G08940, AT3G21055, AT1G67740, AT1G03130, AT2G45180, AT4G35770, AT4G01935, AT5G16930, AT2G34420, AT1G15820, AT2G34420, AT4G10340, AT1G61520, AT2G06520, AT3G27690, AT3G15190, AT1G77680, AT3G07770	[18, 63, 66, 70]
Regulation of cell cycle or differentiation	AT5G66570, AT2G26650, AT5G46070, AT2G42840	[18]
Transporter	AT1G55670, AT1G03130, AT2G45180, AT4G11670, AT5G66570, AT3G11964	[18]
Regulation of protein dephosphorylation or other modification	AT3G08940, AT3G21055, AT1G67740, AT1G03130, AT1G15820, AT5G12400, AT2G20850	[18]
Protein synthesis or proteolysis	AT3G15190, AT1G77680	
Nucleotide binding, regulation of transcription	AT3G08940, AT3G07650, AT5G66570, AT5G12400, AT2G42270, AT1G70060, AT2G40770, AT5G04290, AT1G33700, AT4G35240, AT1G14790	[18]
sn (o) RNA or other ncRNA	AT4G13495, AT5G09585	
ATP binding, ATPase or kinase activity	AT2G20850, AT2G42270, AT2G40770, AT1G17750	
Signaling transduction	AT5G22690, AT2G20850, AT5G13000, AT1G64060, AT1G17750	[18, 71]

^a^Suggests the function of RNA itself, for example, rRNA, or the functions in its expressed proteins

^b^The functions of many transcripts were inferred by gene ontology (GO) analysis using the online tool in TAIR (http://www.arabidopsis.org/) and some functions were inferred from the recent publications

**Table 7:** Potential functions of the 41 transcripts presenting extensively higher m^6^A methylation in flowers than that in leaves and roots in *Arabidopsis* (fold change >10 or <0.1, FDR <10^−10^)

Potential functions^a^	Gene ID	References^b^
Transporter	AT1G15960, AT3G16460, AT1G80270, AT4G20860, AT3G23560, AT4G18197, AT5G44110, AT3G04620, AT4G39100	[72–75]
Defense or stress response	AT2G02100, AT1G27170, AT4G25720, AT3G16460, AT2G14080, AT4G39100, AT4G20860, AT3G23560, AT2G01830	[73, 76–78]
Redox process	AT3G50440, AT4G34900, AT2G07785, AT4G36530, AT4G20860	[18, 79]
Differentially or specifically expressed during flowering	AT3G23450, AT1G44890, AT5G44110, AT1G18370, AT1G05070, AT3G04620, AT5G62580, AT2G27380, AT2G45730, AT3G23560,	[18, 80–83]
Response to abscisic acid stimulus	AT1G76260	[84]
Cell proliferation, differentiation or circadian rhythm	AT1G78910, AT1G18370, AT1G19990, AT3G04620, AT5G62580, AT2G45730, AT5G66550, AT2G05440	[18, 80, 82]
Protein located in mitochondria or chloroplast	AT1G78910, AT4G25720, AT5G22608, AT1G80270, AT3G01200	[70, 85]
Nucleotide binding, regulation of transcription	AT1G80270, AT2G45730	
Protein synthesis, modification, or proteolysis	AT3G28500, AT3G27110, AT2G40205, AT2G23890, AT2G45730, AT1G07070, AT4G05040, AT2G01830, AT2G27900,	[18, 86, 87]
ATP binding, ATPase or kinase activity	AT3G01200, AT2G14080	
Signaling transduction	AT2G27900, AT2G14080, AT2G01830	[78, 88]
Carbohydrate metabolism or energy release	AT3G22210, AT1G22940, AT4G36530	[18]
sn (o) RNA or other ncRNA	AT3G57645	

^a^Suggests the function of RNA itself, for example, rRNA, or the functions in its expressed proteins

^b^The functions of many transcripts were inferred by gene ontology (GO) analysis using the online tool in TAIR (http://www.arabidopsis.org/) and some functions were inferred from the recent publications

**Table 8:** Potential functions of the 23 transcripts presenting extensively higher m^6^A methylation in roots than that in leaves and flowers in *Arabidopsis*

Potential functions^a^	Gene ID	References^b^
Transporter	AT5G52050, AT4G27140, AT4G29030, AT4G28520	[18]
Nutrient reservoir activity	AT4G27140, AT1G03890, AT4G28520	[89] Gruis
Cell proliferation, differentiation or [AQ6]	AT2G36120, AT1G56660, AT4G34410, AT2G22860,	[81, 90, 91]
Defense or stress response	AT4G34410, AT5G07010, AT5G52050, AT2G05940, AT5G42380, AT4G28520	[18, 92–96]
Redox process	AT4G34410, AT1G18300, AT4G12960, AT2G34600	[97]
Carbohydrate metabolism	AT1G27440	[98]
Regulation of transcription	AT4G34410	
Protein located in mitochondria or amyloplast	AT1G18300	
Protein dephosphorylation or phosphorylation	AT1G48040, AT4G18250, AT5G42370	
Signaling transduction	AT4G34410, AT5G07010, AT2G05940, AT2G34600, AT4G18250, AT4G28520	[18, 99, 100]
Unknown protein	AT1G62080, AT1G62000, AT1G62333	

^a^Suggests the function of RNA itself, for example, rRNA, or the functions in its expressed proteins

^b^The functions of many transcripts were inferred by gene ontology (GO) analysis using the online tool in TAIR (http://www.arabidopsis.org/) and some functions were inferred from the recent publications

### Gene transcript level and m^6^A RNA methylation patterns in the transposable element genes

**Fig. 10:**
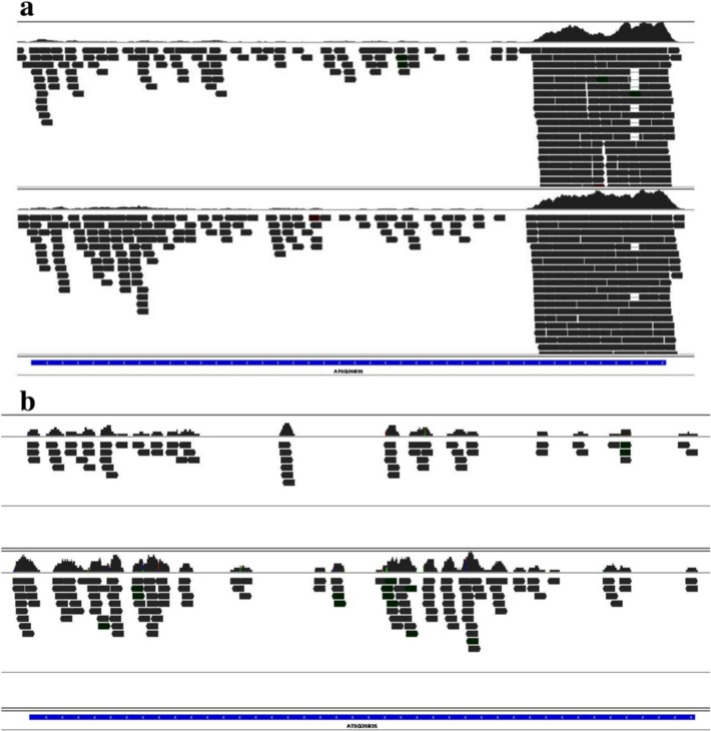
The transcriptional pattern (**a**) and m^6^A patterns (**b**) in the TE transcripts. The ‘fragmented form’ was observed in most of the TE transcript (the representative gene, AT5G35835, and trace files of two organs (leaves and flowers))

Interestingly, more than 97 % of the transcripts from the transposable element gene (TE) exhibited relatively high extent of m^6^A modification compared to gene transcript level (fold change of NFPKM between two organs >2 or <0.5, FDR <0.05). Another intriguing phenomenon was that most (>75 %) of the TE transcripts presented a ‘fragmented’ form in the both m^6^A-seq and mRNA-seq data (Fig. 10). The transcript level was distinct among the fragmented TE transcripts, though the transcript fragments were derived from the same TE in the genome (Fig. 10a). In some cases, the 5′ region of TEs was highly transcribed, but the other regions were much less expressed (Fig. 10a). Therefore, most of the TE transcripts remained a ‘fragmented’ form in the cells with a relatively high m^6^A extent and a low transcript level.

## Discussion

### Improvement of biotechnology is prerequisite for successful m^6^A-seq

RNA isolation from plants is sometimes difficult due to a thick cell wall which may be difficult to disrupt during RNA extraction [101, 102]. In addition, plant cells also produce secondary molecules (for example, polysaccharides), which are collected during RNA precipitation and can be problematic for RNA purification [101]. We modified the CTAB protocol for RNA isolation. The improved buffer showed a potential to efficiently disrupt cell walls, and specifically precipitate RNA, thus a large quantity of high purity RNA from *Arabidopsis* was obtained for mRNA-seq, m^6^A-seq, and input RNA-seq in this study (Additional file 11).

Another significant improvement in this study is that we optimized the protocol for m^6^A RNA immunoprecipitation to improve RIP efficiency and to minimize non-specific precipitation. We found that procedures including pre-precipitation through adding beads and IP buffer in the RNA solution before adding m^6^A antibody to exclude any non-specific binding RNA, and a vigorously washing of the binding beads after incubation with m^6^A antibody three to four times using IP buffer can significantly reduce the background (non-specific precipitation) in the RIP experiment. Incubation of the beads binding with RIP RNA in the dilution buffer at 50 °C for 90 min facilitated washing down of m^6^A RNA from the beads to improve m^6^A RNA yield.

### m^6^A patterns between plant and mammal

The m^6^A patterns between plant (Figs. 4a and 5) and mammal were similar, for example, both rich near stop codons and 3′UTR [6, 8, 12, 13]. Both kingdoms had a similar consensus m^6^A methylation motif ‘RRm^6^ACH’ and a close frequency of m^6^A site per transcript in the transcriptome [6, 12, 13]. These phenomena indicated that m^6^A RNA methylation may be conserved between plant and mammal.

Some differences, however, were found between *Arabidopsis* and mammals as aforementioned (for example, Fig. 3a). These differences more likely represented the different m^6^A methylation extent and unique patterns between two species, or may result from the distinct depths of m^6^A-seq between two studies. Resolution in the m^6^A-seq with a low depth in the previous studies may be insufficient to discern the weak m^6^A signals in the coding regions in most of mRNA (Figs. 4a and 5). The previous report also suggested under-estimation of m^6^A sites in their studies [6, 8].

Dominant m^6^A enrichment in 5′UTR in *Arabidopsis* was reported by Luo *et al.* [13]. However, both our study from six *Arabidopsis* m^6^A-seq datasets (Fig. 5) and a recent report from rice m^6^A-seq of two different tissues [12] did not observer this dominant m^6^A pattern as the authors claimed a unique m^6^A pattern in the plant in their paper [13]. The m^6^A modified motif was confirmed by numerous studies highly conserved among the eukaryotes, including between two kingdoms of plant and animal [2, 6–8, 103]. A number of studies did not observe the dominant m^6^A enrichment in 5′UTR in mammals either [6, 8, 11]. The dominant m^6^A near the start codon reported by Luo *et al*. [13] may be an experimental artifact, for example probably caused by contamination from mRNA as a dominant peak was also found near the start codon in the overall pattern of mRNA-seq (Fig. 5). Nevertheless the difference in m^6^A enrichment pattern needs further confirmation.

The proportion of the m^6^A modified transcripts (over two-third) estimated in this study is relatively much higher than the previously reported [6, 12]. The previous studies with the robust experiments confirmed that the m^6^A modification is required for RNA stability and RNA transport from nucleus to cytoplasm [4, 6]. Otherwise, RNA will be degraded in the cells [3, 4, 6]. This suggested that the major proportion of mRNA in the cells, for example, over 70 % as estimated in our study, may rationally be modified by m^6^A [3]. Therefore, our estimation may reflect the genuine phenomenon in the cells.

### Extensively high m^6^A methylation in certain transcripts may be suitable or required for the biological functions of these transcripts

Eukaryotic cells are highly compartmentalized and functionally differentiated [104–106]. mRNA translated into proteins for energy carriers (for example, ATP or GTP), transporter (for example, ion transporter), stress response, redox, protein post-translational modification, and protein located in mitochondria or chloroplast were found highly methylated by m^6^A in this study (Table 3). The common feature of these proteins was that they were a direct transporter for energy molecules, for example, ATP or GTP, or accomplishment of their biological functions was dependent on these energy chaperones [106, 107]. These transcripts may be transported for their protein translation near compartments for energy metabolism in the cells (for example, near mitochondria or chloroplast) or in the cells which were highly differentiated for energy process or stress response [109–111]. A long evolutionary history of m^6^A modification may imprint these transcripts with extensively high m^6^A methylation for their molecule stability [4, 112].

Extensive high m^6^A methylation was also found in the transcripts for some signaling factors, regulation factors (for example, regulation of DNA replication, RNA transcript, and protein synthesis and post-transcriptional process) and in certain sn(o)RNA (Table 3). These transcripts were highly or delicately regulated to maintain a low TL in the cells in most cases [107, 108, 113]. High methylation in these transcripts may endow RNA stability to these transcripts with a low TL, or confer signaling recognition to these transcripts [1, 3, 6, 112].

The transcripts of certain pseudogenes were extensively modified by m^6^A (Table 3). Accordingly, the transcripts of their relative functional genes had high methylation, for example, gene for NADH dehydrogenase (‘AT2G07709’) (Table 3). m^6^A modification proved a highly selective and regulative event [6, 8, 112]. If the transcript of a parent functional gene was extensively methylated by a regulative system, the transcript of the relative pseudogene may also be highly methylated by the same regulative system.

Dysfunction of m^6^A modification in the human cells resulted in an abnormal circadian rhythm [11]. Several transcripts responsible for regulation of circadian rhythm were highly methylated by m^6^A in *Arabidopsis* (Tables 3 and 7), suggesting that m^6^A may also play an important role in regulation of circadian rhythm in plants.

### The m^6^A topological patterns and their potential functions in different types of RNAs

A frequency of 15 consensus (RRm^6^ACH) sequences per transcript should occur in the transcriptome [2, 14]. In deed, the occurrence of m^6^A methylation in the transcriptome was much lower than this expected frequency [2, 6, 114], suggesting that the majority of the consensus sequences were either not modified or many of them were demethylated promptly after the accomplishment of the biological metabolisms due to m^6^A modification [2]. As a result, methylation and demethylation may maintain a dynamic oscillation in the cells in response to environmental stimuli [2, 115, 116].

The extensively lower m^6^A signals in the coding regions including the splicing sites in most of mRNA discovered in this study (Figs. 4a and 5) could come from the sequencing bias. But the technical bias resulting in a large number (over 70 %) of the m^6^A modified mRNA containing this feature is impossible (Fig. 4a). The m^6^A methylation is required for a correct RNA splicing event [6, 116, 117]. Nevertheless, the most possible reason for the low signal detected in the coding regions in most of mRNA in this study may result from a prompt m^6^A demethylation event after accomplishment of the RNA splicing event [115, 116]. The consequent m^6^A demethylation in the coding regions may facilitate a speed of movement of ribosomes through mRNA chain thus may confer a high efficiency protein synthesis, while reservation of a great higher extent of methylation at the stop codon or 3′UTR may be responsible for RNA stability, signaling for transport and translocation, or as regulatory elements for protein translation through the recruitment of specific factors onto these m^6^A sites for RNA transport or protein synthesis [4, 112, 118, 119].

Dominant m^6^A enrichment near stop codons and 3′UTR (as shown in Fig. 4a) was observed in most (over 70 %) of mRNA in this study as previously reported [6, 8, 12, 13]. This m^6^A distributing type (Fig. 4a) may represent the typical m^6^A topological pattern in most of the mature mRNA. However, a small proportion of mRNA did not present this m^6^A enrichment in 3′UTR (as shown in Fig. 4b). m^6^A methylation confers RNA stability [4]. Non-dominant m^6^A enrichment in 3′UTR in this small proportion of mRNA may reflect a phenomenon of ‘subsequent-demethylation’ to the m^6^A dominant peaks in 3′UTR or stop condon, further suggesting that these RNA may undergo a process of degradation due to m^6^A demethylation [4]. Therefore, different m^6^A topological patterns in mRNA may reflect RNA status or fate in the cells, for example, pre-mature RNA, mature RNA, or RNA being in degradation.

Both rRNA and tRNA were mainly modified by numerous types of cytosine methylation [1, 3]. However, a relatively high m^6^A methylation was found in all rRNAs (Fig. 6a and b) and in a certain proportion of tRNAs (approximately 10 %) (Fig. 6c) in *Arabidopsis* in this study. Mature rRNA and tRNA were derived from pre-rRNA and pre-tRNA through a series of complex biological and molecular processes, including splicing and folding. The splicing machinery in rRNA and tRNA was similar to that in mRNA in eukaryotes [3]. m^6^A may guide a correct splicing event in these types of RNAs as the role of the m^6^A methylation required for the correct splicing events in mRNA [6]. m^6^A may not undergo a subsequent demethylation event after splicing for the mature rRNA, but demethylation of m^6^A after splicing may be required for the mature tRNA as hypothetically aforementioned for most of mRNA. This would be the reason that m^6^A methylation was reserved and observed in all rRNAs (Fig. 6a and b), but it was detected in a small proportion of tRNAs (Fig. 6c).

Therefore, diverse m^6^A patterns and topologies may be unique or required for the miscellaneous functions in the different types of RNAs [3, 112].

### Potential roles of differential m^6^A methylation among plant organs

Differential gene expression among plant organs has proved responsible for organ differentiation and development [120, 121]. Differential level of m^6^A methylaytion among three organs was much higher than that in gene transcript level (Table 4), suggesting that m^6^A modification may be another important contributor for organ differentiation or maintenance of differential status among the organs in *Arabidopsis*.

The transcripts presenting extensively higher m^6^A modification in an organ than other organs showed a connection of the functions of these transcripts required for or specific to this organ. For example, the transcripts presenting an extensively higher level of m^6^A methylation in leaves were related to photosynthesis metabolism or proteins located in mitochondria or chloroplast, and those in roots showed response to stresses, redox process, and transporters (Tables 6 and 8). However, most of the differential and extensively methylated transcripts in flowers were related to regulation of reproductive organ development, stress response, cell proliferation, differentiation, or circadian rhythm (Table 7) [18, 122]. Dysfunction of *METTL3*, the gene responsible for m^6^A modification, can result in an arrest of the early development in embryo at the globular stage in *Arabidopsis* [7], suggesting that m^6^A methylation may play an important role in differentiation and development of the reproductive organs in plants.

The proportion of the m^6^A modified transcripts was the highest in the roots among three organs (Additional file 3). The surrounding environment for root growth is more complex than that for leaves and flowers [123, 124], which may require a higher proportion of the m^6^A transcripts in roots to adapt to more diverse conditions [2–4]. However, the overall extent for m^6^A RNA methylation showed the highest in the leaves among three organs (Table 1). The leaves are the major organ in plants responsible for photosynthesis metabolism and energy transition [123]. This may require a higher extent of m^6^A RNA methylation in the leaves to fit for these metabolisms that were processed in severe conditions in most of cases if our hypothesis aforementioned is rational.

### Potential biological significance of gene transcript and m^6^A patterns in TE

Most of the TE transcripts (>85 % in this study) remained a relatively low level in the cells. This may repress a neo-transposition of TEs into the genome [125]. 17 % to 85 % of the genomic sequences are composed of TE in the higher plant species [126, 127]. In most of cases, the neo-transposition of TEs was highly repressed because it is often detrimental to the host due to a prompt expansion of the genomic size or dysfunction of the functional genes by this event [128]. The fragmented TE transcripts may not be reversely transcribed into a complete cDNA, thus the malignant TE transposition may be consequently avoided. In addition, the distribution of these unconnected transcripts in the cells may play a role similar to the small interfering RNA (siRNA), which may in turn repress the transcription or the neo-transposition of these TEs [125, 129]. The remnant TE transcript fragments may also be induced in response to stress, which may further trigger a serial of complex reactions [127]. Some of the fragmented TE transcripts may play a role as ‘a regulator’ for other gene expression or as a direct regulator for the nearby genes [125, 127, 130, 131], or may work as powerful regulators of the immune response as the functions recently discovered in some miRNA [132]. Because the fragmented transcripts were more likely to be degraded than the intact transcripts [3], relatively high m^6^A methylation in the fragmented TE transcripts may prevent these segmented transcripts from further degradation so that a relatively low level of these transcripts can be maintained in the cells [3].

## Conclusions

Thanks to significant improvements of technologies for RIP experiments in this study, high resolution of transcriptome-wide mapping of m^6^A was available in *Arabidopsis*. This is the first study for comprehensive characterization of m^6^A patterns of different types of RNAs, relationship between m^6^A methylation extent and gene transcript level, and differential features of m^6^A methylation among three plant organs. Two-third of the transcripts were modified by m^6^A in *Arabidopsis*. 35,000 to 48,000 m^6^A sites and approximately 1.4 to 2.0 m^6^A sites per transcript were mapped to the *Arabidopsis* genome.

Over 85 % of the m^6^A modified transcripts had a relatively high m^6^A methylation level (*P* <0.05), while <4 % had a low m^6^A extent compared with their transcript level (*P* <0.05). Approximately 5.5 % of the methylated transcripts presented extensively high methylation (fold change >10 compared to TL, and FDR <10^−12^). The highly expressed transcripts were relatively less methylated by m^6^A and *vice versa* (*P* <0.001). The 290 (2.1 %) highly methylated transcripts were mainly expressed for stress response, redox, signaling factors, regulation factors, and some ncRNA. Most of the biological functions in these transcripts were involved in molecule binding, transferase, transporter, and kinase activity.

Most of mRNA (over 70 %) was characterized by a typical m^6^A topology, that is, one or two predominant peaks at the stop codon or 3′UTR accompanying with extensively low m^6^A signals in the coding regions. Unlike a recent observation of another predominant m^6^A enrichment in the 5′ mRNA in *Arabidopsis* [13], we found that m^6^A predominantly distributed only at the stop codon or 3′UTR. Some sn(o) RNAs were also highly methylated with a single m^6^A site in these transcripts. All rRNAs was relatively highly methylated by one or several m^6^A sites. Non- or slight-m^6^A methylation was observed in most of tRNAs (approximately 90 %), and the remaining tRNAs were relatively hyper-methylated by a single m^6^A site. Interestingly, most (over 75 %) of the transcribed TEs maintained a relatively high m^6^A methylation. Therefore, the topologies of m^6^A in different RNAs not only confer diverse m^6^A patterns in the cell, the unique m^6^A pattern in a specific RNA may endow special functions to this RNA.

The similar m^6^A patterns between plant and mammal suggested that m^6^A methylation may be conserved between two living kingdoms. Differences were also found between plant and mammal, which may represent their unique m^6^A patterns in the two living kingdoms.

Proportion of transcripts (33.5 %) showing differential m^6^A methylation among three organs was greatly higher than that (22.6 %) presenting differential transcript level in *Arabidopsis* (*P* <0.00035). Function of the transcripts with extensively higher methylation in an organ than others were required or suitable for unique biological roles of this organ. Therefore, m^6^A methylation may be an important contributor to the organ differentiation and may confer unique functions to this organ.

## Methods

### Plant materials

Wild type, Columbia ecotype (Col-0), of the mouseear cress (*Arabidopsis thaliana*) was used in this study. The plants were grown in the greenhouse under a photoperiod of 16 h light/8 h dark at 22–24 °C. When the plants were at the blooming period (5 weeks after seed germination), the plant materials of the flowers, rosette leaves, and roots were separately collected, treated with liquid nitrogen and stored at −80 °C until use.

### RNA isolation and purification

All centrifuge tubes and pipette-tips are RNase-free or must be treated with DEPC. All buffers were RNase free or prepared using DEPC-treated dd H_2_O. The modified CTAB buffer was used for RNA isolation [133]. In brief, approximately 10 g of the frozen plant materials were ground into fine powder in liquid nitrogen. The plant powder was promptly transferred into four 50 mL tubes with 25 mL CTAB buffer (2 % CTAB, 1.0 % PVP-40, 2.0 M NaCl, 100 mM Tris, 25 mM EDTA-Na_2_, and 1.0 % β-mercaptoethanol) in each tube. Incubate the tubes at 65 °C for 10 min and invert tubes for several seconds every minute during incubation. Add 200 μL chloroform to each tube, invert quickly 150–200 times. Centrifuge tubes at 4,500 g for 2 min at 4 °C, and discard most of the chloroform solution using a pipette-tip. Centrifuge tubes at 15,000 g for 5 min at 4 °C. Transfer the supernatant to new tubes. Add 8.0 mL LiCl (8.0 M) into each tube and mix well. Store the mixture at −20 °C for 30 min, then centrifuge at 17,000 g for 30 min at 4 °C. Use 2 mL 80 % ethanol to rinse RNA pellet. Centrifuge at 17,000 g for 2 min, discard the ethanol, and then dry tubes in the laminar-flow hood. Dissolve the dried RNA pellet using 400 μL RNase-free water and treat with DNase (Promega, Madison, WI, USA) to remove DNA contamination. Add 300 μL chloroform-phenol (1:1, v/v) into the tube, invert quickly 200 times. Centrifuge tubes at 13,000 g for 1 min at 4 °C, and discard most of the chloroform-phenol solution in the hood. Centrifuge tubes at 15,000 g for 5 min at 4 °C. Transfer the supernatant to new tubes. Add 2.8 volume of ethanol and 0.10–0.15 volume of NaAc (pH 5.6), and mix well. Store the mixture at −20 °C for 30 min, then centrifuge at 15,000 g for 30 min at 4 °C. Store the RNA pellet in the ethanol-NaAc solution at −80 °C until use.

### RNA fragmentation

The purified total RNA was diluted in the fragmentation buffer (10 mM ZnCl_2_ and 10 mM Tris–HCl, pH 7) with the RNA final concentration of approximately 1.0 μg μL^−1^ [6, 10]. The diluted RNA was fragmented into approximately 100-nucleotide-long by incubation at 95 °C for 5 min. The chemical fragmentation reaction was stopped with 0.05 M EDTA. The ethanol-NaAc solution was used to precipitate RNA. The fragmented RNA was prepared for RIP and m^6^A-seq.

### RNA immunoprecipitation (RIP)

Approximately 2.5 mg fragmented total RNA was resuspended in the 1,000 μL IP buffer (150 mM NaCl, 0.1 % NP-40, 10 mM Tris–HCl, pH 7.4, 2 mM RVC (Sigma-Aldrich, St. Louis, MO, USA), 200 U RNasin (Promega, Madison, WI, USA), and 0.5 mg mL^− 1^ BSA). Non-specific binding RNA was pre-precipitated by adding 3.0 μL Protein A bead (Life Technology, Grand Island, NY, USA) and incubation on a rotating wheel for 2 h. The magnet rack was used to precipitate the beads. The supernatant was transferred to a new tube. Add 5.0 μg m^6^A-specific monoclonal antibody (Merck Millipore, Billerica, MA, USA) into the tube containing RNA and IP buffer, and incubate on a rotating wheel for 3–4 h. Then add 5.0 μL Protein A bead (Life Technology, Grand Island, NY, USA) for an additional rotation for 2 h. The magnet rack was used to precipitate the beads. The beads were vigorously washed using 1,000 μL IP buffer three to four times. Discard the IP buffer. Add 300 μL dilution buffer (10 mM Tris–HCl pH 7.5) into the bead tube and incubate at 50 °C for 90 min. Precipitate the beads using the magnet rack and transfer the supernatant to a new tube. The ethanol-NaAc solution and glycogen were used to precipitate m^6^A RNA. The m^6^A RNA pellet was washed using 80 % ethanol and then resuspended into 15 μL dd-H_2_O for m^6^A-seq and HPLC-MS/MS analysis.

### mRNA-seq, m^6^A-seq, and RNA-seq from the input samples

The quality control (QC) tests for the RNA samples were performed using Agilent Technologies (Santa Clara, CA, USA). Library for mRNA-seq was generated using mRNA sequencing kit plus random primers. High throughput m^6^A-seq, mRNA-seq, and input RNA-seq of three samples of leaves, flowers, and roots was performed on HiSeq 2000 (Illumina Inc., San Diego, CA, USA) at Purdue University Genomics Core Facility (http://www.genomics.purdue.edu/services/core.shtml).

Approximately 10.0 μg purified total RNA was reserved for mRNA-seq before the RNA samples were used for fragmentation. And approximately 2.5 μg fragmented total RNA was reserved and used for the input RNA-seq before the RIP experiments. Thus, mRNA-seq, m^6^A-seq, and input RNA-seq were parallel and their data were mutually comparable in this study [6]. RNA integrity number (RIN) was estimated using a Nanodrop 2000 UV vis (Thermo Fisher Scientific, Wilmington, NC, USA). The QC tests were done by Agilent Technologies (Santa Clara, CA, USA). All RNA sequencing of three samples of leaves, flowers, and roots was performed on the same sequencer at the same batch.

### HPLC-MS/MS

A total of 100–200 ng of input total RNA or m^6^A RNA from RIP experiment was digested by 2 U nuclease P1 (US Biological Life Science, Salem, MA, USA) at 37 °C for 2 h, and 0.5 U alkaline phosphatase (Promega, Madison, WI, USA) at 37 °C for an additional 2 h. A total of 5 μL of the digested and purified solutions were assayed by HPLC-MS/MS. Nucleosides were separated by reversed-phase high performance liquid chromatography, using a Waters Xterra C18 column (2.1 × 150 mm, 3.5 μm) with a water/acetonitrile gradient. Mass spectrometry was performed using an Agilent 6460 triple quadrupole mass spectrometer in positive electrospray ionization mode. Mass transitions were 268.1 -- > 136.1 for A and 282.1 -- > 150.1 for m^6^A (Additional file 2).

### Alignment of reads and visualization of m^6^A peaks

Both RNA-seq and m^6^A-seq datasets were mapped to the *Arabidopsis* genome (TAIR10) using TopHat2 with a parameter of ‘-b2-fast’ [134]. The potential PCR duplicates were removed by the parameter ‘rmdup’ rooted in SAMtools [135]. The fragment numbers for each transcript were estimated using the featureCounts with a parameter of ‘-p’ [136].

The peaks and distributing patterns of m^6^A in the *Arabidopsis* transcriptome were visualized using free software, Integrative Genomics Viewer (IGV2.3, Boston, MA, USA [137]).

We tried several previously published protocols, for example, moving-window, to call m^6^A peaks based on comparison of m^6^A-seq with input RNA-seq counterpart. And we found all of these methods failed recognition of most (>92 %) of m^6^A sites in CDS (Additional file 12). We finally did not apply these methods in this study. Because of the extensively low non-specific immunoprecipitation rate (<1 %) in this study (Additional file 2), all the mapped reads in the m^6^A-seq were considered to be sequenced and derived from the specific immunoprecipitation of the m^6^A RNA fragments. Thus, an estimation of m^6^A peak number of a m^6^A modified transcript was calculated by this formula: total mapped absolute length covered by m^6^A fragments within the transcript/150, considered that library construction for m^6^A-seq was created from a m^6^A RNA pool with an average RNA length of approximately 106 nucleotides (Additional file 11: Fig. S3b) and average coverage of a peak in the m^6^A-seq data base was approximately 150-nucleotide long in this study as visualized by IGV 2.3.

### Discernment of m^6^A topological patterns

Distribution of m^6^A sites in the different regions of the transcripts was estimated by Dominissini *et al.*’s method [6]. The consensus m^6^A motif sequences were figured out by Luo *et al.*’s protocol [13] with modification: approximately 1,000 the highest m^6^A peaks and approximately 100 nt length around each m^6^A peak were used for deduction of the consensus m^6^A motif sequences. The typical m^6^A patterns of different types of RNA were drawn from the m^6^A mapping analysis based on visualization using the IGV2.3 program [13].

The overall m^6^A distributing patterns were discerned by this method: a gene was splitted in to 60 bins. The read depth of each bin was normalized by per 1 kb per 1 Mb data, then the normalized depth was used to plot the patterns.

### Comparison of m^6^A methylation extent versus transcript level

The sequenced fragment number of each transcript in mRNA-seq was normalized using the algorithm of Fragments Per Kilobase of Transcript Per Million Fragments Mapped (FPKM = Counts of mapped fragments × 10^9^)/(Length of transcript × Total count of the mapped fragments); ‘fragment’ refers to mapped reads after removal of PCR duplicates [138]. While the sequenced fragment number of each transcript in m^6^A-seq was normalized using a modified FPKM (MFPKM = Counts of mapped fragments × 10^9^)/(Total absolute mapped length on the chromosome covered by m^6^A fragments within the transcript × Total count of the mapped m^6^A fragments)), considered that the library in m^6^A-seq was derived only from RNA fragments containing m^6^A sites, and not from the entire transcript [138].

The m^6^A methylation extent of a transcript were categorized into three groupings based on comparison of MFPKM of the transcript in the m^6^A-seq with the FPKM of the same transcript in the mRNA-seq using *χ*^2^ test: (1) m^6^A methylation extent ‘equivalent’ to transcript level (‘equivalent’, ratio of FPKM to MFPKM fits 1:1 (*P* <0.05)); (2) methylation extent higher than transcript level (‘Hi’, ratio of FPKM to MFPKM < 1 (*P* <0.05)); and (3) methylation extent lower than transcript level (‘Low’, ratio of FPKM to MFPKM >1 (*P* <0.05)). The chi-square value was determined by this formula: *χ*^2^ = (MFPKM*100/(MFPKM + FPKM) - 50)^2/50 + (FPKM*100/(MFPKM + FPKM) - 50)^2/50.

### Analysis of both differential transcript level and differential m^6^A methylation among plant organs

mRNA-seq data were normalized by FPKM as described above. *χ*^2^ tests were used to estimate whether FPKM was significantly different between two organs using R 3.1 (http://cran.r-project.org/bin/windows/base/). The transcripts with fold change in FPKM >2.0 or <0.5, and FDR <0.02 were considered differentially expressed between two organs [6, 10].

To minimize influence of transcript level on estimation of differentiation of m^6^A extent, m^6^A-seq data were normalized by a specific algorithm, NFPKM (NFPKM = MFPKM in m^6^A-seq/LOG (FPKM in mRNA-seq, 2)). *χ*^2^ tests were also used to estimate whether NFPKM of a m^6^A modified transcript was significantly different between two organs using R 3.1. The transcripts with fold change of NFPKM >2.0 or <0.5, and FDR <0.005 were considered differentially methylated between two organs [6, 10]. The transcripts commonly presenting higher m^6^A extent in an organ in the two replicates were used for gene ontology (GO) analysis. *P* values of the chi-square tests to all gene transcripts of a replicate were input in R 3.1.2 (‘Pumpkin Helmet’) to estimate FDR of each transcript under a parameter of ‘p.adjust (*P* value,method = ‘fdr’)’. The fold change is a fixed threshold for all classifications, thus FDR thresholds may vary with different analyses.

### Visualization of the overall differential patterns

The overall patterns of differential transcript level of all transcripts were discerned using ratio of FPKM of mRNA-seq between two organs. Similarly, the overall patterns of differential m^6^A methylation of all transcripts were depicted using ratio of NFPKM derived from both m^6^A-seq and mRNA-seq (see above) between two organs. The heatmap representing the overall differential patterns of gene transcript and m^6^A methylation was created using R3.1 (http://cran.r-project.org/bin/windows/base/).

### GO and KEGG pathway analysis

The GO results and potential molecular functions in the transcripts were deducted from the online tool released in the TAIR website (http://www.arabidopsis.org/) [139]. And molecular functions in some transcripts were inferred from the recent publications.

The Kyoto Encyclopedia of Genes and Genomes (KEGG) pathways in certain transcripts of interests were figured out using the online tool released in the DAVID bioinformatics resources (http://david.abcc.ncifcrf.gov/).

### qRT-PCR

Quantitative real-time PCR (qRT-PCR) was performed to assess the relative abundance of m^6^A RNA in the RIP samples. All purified RNA templates were transferred into cDNA using Quanta qScript™ cDNA Synthesis Kits (Quanta BioSciences, Inc., Gaithersburg, MD, USA). Eleven genes were randomly chosen for this test (Additional file 9). The qRT-PCR primers were designed to span exon-exon junctions in order to eliminate the potential amplification of genomic DNA and non-spliced mRNA. qRT-PCR was performed on C1000 Thermal Cycler (Bio-RAD) using SYBR Green SuperMix buffer (Bio-RAD) and 300 ng total cDNA template for amplification. Because the qRT-PCR amplicon spanned an exon-exon junction with a length of 80–150 bp and the m^6^A enrichment in the region around splicing sites was usually low in most of mRNA in this study (Fig. 4a), cDNA from total RNA of the *Actin2* gene was used for housekeeping gene to estimate the relative abundance (RA) of m^6^A RNA in the qRT-PCR amplicons based on this algorithm: RA = 100 × 2^−ΔC^. The expected abundance (EA) of m^6^A RNA in the m^6^A-seq dataset was estimated by this algorithm: EA = 100× (the mapped m^6^A RNA reads of the test gene in m^6^A-seq and in the region for qRT-PCR test/ the mapped RNA reads of the *Actin2* gene in mRNA-seq and in the region for qRT-PCR test). The consistency between the AR and ER patterns among three organs was compared (Additional file 10).

### Ethics statement

The plant materials used in this study are freely used and available to all researchers without any protection for intellectual property right. This research meets all applicable standards for the ethics of experimentation and research integrity from all five institutes that provide supports to this study.

### Data access

The data discussed in this publication have been deposited in NCBI’s Gene Expression Omnibus and are accessible through GEO Series accession number GSE72706.

## Additional files

Additional file 1: Table S1. The sequenced and mapped reads in the m^6^A-seq, mRNA-seq, and input RNA-seq samples. (DOC 43 kb) Additional file 2: Figure S1. The m^6^A peak and adenosine peak deduced from the HPLC-MS/MS analysis. **a** The relative m^6^A peak height (upper) and adenosine peak height (lower) in the standard sample. **b** The relative m^6^A peak height (upper) and adenosine peak height (lower) in the input sample. **c** The relative m^6^A peak height (upper) and adenosine peak height (lower) in the RIP sample. (DOC 50 kb) Additional file 3: Table S2. Number of transcripts in the mRNA-seq and the m^6^A-seq (RIP) samples and proportion of the m^6^A modified transcripts in the three organs of *Arabidopsis*. (DOC 33 kb) Additional file 4: Table S3. Number of m^6^A sites detected in the three organs of *Arabidopsis*. (DOC 37 kb) Additional file 5: Table S4. Ratio of m^6^A/A in the three organs of *Arabidopsis*. (DOC 38 kb) Additional file 6: Table S5. Category of the m^6^A modified transcripts based on the number of m^6^A sites per transcript. (DOC 37 kb) Additional file 7: Table S6. Proportion of two types of m^6^A distributing feature in mRNA. (DOC 30 kb) Additional file 8: Table S7. The transcriptome-wide normalized read depth in the 60 bins of the gene representing the overall m^6^A patterns in the different regions of the genes. Non-significant differences were found between three organs (*P* = 0.761). (DOC 108 kb) Additional file 9: Table S8. The primers used for qRT-PCR. (DOC 35 kb) Additional file 10: Figure S2. The relative abundance (RA) of m^6^A RNA deduced from qRT-PCR and the expected abundance (EA) of m^6^A RNA deduced from the m^6^A-seq dataset. **a** RA for ‘AT1G35710’, **b** EA for ‘AT1G35710’, **c** RA for ‘AT3G07610’, **d** EA for ‘AT3G07610’, **e** RA for ‘AT4G14410’, **f** EA for ‘AT4G14410’, **g** RA for ‘AT2G28490’, **h** EA for ‘AT2G28490’, **i** RA for ‘AT1G03880’, **j** RA for ‘AT1G03880’, **k** RA for ‘AT1G33700’, **l** EA for ‘AT1G33700’, **m** RA for ‘AT2G07836’, **n** EA for ‘AT2G07836’, **o** RA for ‘AT5G20960’, **p** EA for ‘AT5G20960’, **q** RA for ‘AT5G22700’, **r** EA for ‘AT5G22700’, **s** RA for ‘AT3G13400’, **t** EA for ‘AT3G13400’, **u** EA for ‘AT4G38120’, **v** EA for ‘AT4G38120’. (DOC 1342 kb) Additional file 11: Figure S3. RNA QC results of the total RNA and the RIP RNA for m^6^A-seq samples. **a** RNA quality for the total RNA sample was high with RIN over 8.5. **b** RNA fragmentation for the m^6^A-seq samples was consistent in the experiments, with an average length of 106 nt. (DOC 313 kb) Additional file 12:
**Spreadsheet files show the m** ^**6**^ **A peaks detected in three organs using ‘Moving-window’ program and their positions in the genome.** (ZIP 547 kb)

## Abbreviations

ATP: adenosine triphosphate
BSA: bovine serum albumin
cDNA: complementary DNA
CDS: coding sequence
CI: confidence intervals
CTAB: cetyl trimethylammonium bromide
DEPC: diethylpyrocarbonate
EA: expected abundance
EDTA: ethylenediaminetetraacetic acid
FDR: false discovery rate
FPKM: fragments per kilobase of transcript per million fragments mapped of the transcript in mRNA-seq
GO: gene ontology
GTP: guanosine triphosphate
HPLC: High-performance liquid chromatography
KEGG: the Kyoto Encyclopedia of Genes and Genomes
m^6^A: *N*^6^-methyladenosine
m^6^A-seq: RNA sequencing based on m^6^A RNA immunoprecipitation
MFPKM: modified fragments per kilobase of transcript per million fragments mapped of the transcript in m^6^A-seq
mRNA-seq: RNA sequencing from oligo-dT RNA
MS: mass spectrometry
NADH: nicotinamide adenine dinucleotide
NFPKM: MFPKM in m^6^A-seq divided by LOG (FPKM in mRNA-seq, 2)
PVP: polyvinylpyrrolidone
QC: quality control test
qRT-PCR: quantitative real-time polymerase chain reaction
RA: relative abundance
RIN: RNA integrity number
RIP: RNA immunoprecipitation
sn(o)RNA: small nuclear RNA and small nucleolar RNA
TAIR: the *Arabidopsis* Information Resource (https://www.arabidopsis.org/)
TE: transposable element gene
TL: transcript level
Tris: tris(hydroxymethyl)aminomethane
TSS: transcription start site
U: unit
UTR: untranslated region
