# Supplementary material for: Transcriptome-wide high-throughput deep m6A-seq reveals unique differential m6A methylation patterns between three organs in Arabidopsis thaliana
Source: Genome Biol. 2015 Dec 14;16:272. doi: 10.1186/s13059-015-0839-2 (PMC4714525; doi:10.1186/s13059-015-0839-2)
Supplement: Additional file 1: Table S1. — The sequenced and mapped reads in the m6A-seq, mRNA-seq, and input RNA-seq samples. (DOC 43 kb) [file 13059_2015_839_MOESM1_ESM.doc]

**Additional file 1: Table S1. The sequenced and mapped reads in the m^6^**A-seq, mRNA-seq and input RNA-seq samples

| Replicate | Samples | Tissues | Category | Clean reads after a removal of adaptors | Cleanly mapped reads |
| --- | --- | --- | --- | --- | --- |
| Replicate 1 | zhwA01 | Leaf | m^6^A-seq | 134,797,106 | 87,455,788 |
|  | zhwF15 | Flower | m^6^A-seq | 115,165,660 | 33,489,846 |
|  | zhwRoot8 | Root | m^6^A-seq | 104,774,124 | 75,518,793 |
|  | zhwLD24 | Leaf | mRNA-seq | 77,905,520 | 73,460,466 |
|  | zhwF15 | Flower | mRNA-seq | 60,349,042 | 57,139,889 |
|  | zhwRoot8CK | Root | mRNA-seq | 92,491,920 | 87,205,832 |
|  | Leaf_rep1 | Leaf | Input, RNA-seq | 51,074,102 | 7,783,758 |
|  | Flower_rep1 | Flower | Input, RNA-seq | 33,603,842 | 19,817,745 |
|  | Root_rep1 | Root | Input, RNA-seq | 31,656,580 | 16,944,034 |
| Replicate 2 | LE03 | Leaf | m^6^A-seq | 90,726,318 | 70,600,665 |
|  | F23 | Flower | m^6^A-seq | 165,894,986 | 125,665,375 |
|  | R07 | Root | m^6^A-seq | 156,536,156 | 125,893,506 |
|  | L1 | Leaf | mRNA-seq | 59,673,962 | 53,807,285 |
|  | F1 | Flower | mRNA-seq | 48,964,626 | 40,562,265 |
|  | R1 | Root | mRNA-seq | 48,106,874 | 39,923,446 |
|  | Leaf_rep2 | Leaf | Input, RNA-seq | 53,643,026 | 9,208,929 |
|  | Flower_rep2 | Flower | Input, RNA-seq | 25,613,866 | 16,322,980 |
|  | Root_rep2 | Root | Input, RNA-seq | 30,648,326 | 16,678,032 |
