# Supplementary material for: Transcriptome-wide high-throughput deep m6A-seq reveals unique differential m6A methylation patterns between three organs in Arabidopsis thaliana
Source: Genome Biol. 2015 Dec 14;16:272. doi: 10.1186/s13059-015-0839-2 (PMC4714525; doi:10.1186/s13059-015-0839-2)
Supplement: Additional file 2: Figure S1. — The m6A peak and adenosine peak deduced from the HPLC-MS/MS analysis. a The relative m6A peak height (upper) and adenosine peak height (lower) in the standard sample. b The relative m6A peak height (upper) and adenosine peak height (lower) in the input sample. c The relative m6A peak height (upper) and adenosine peak height (lower) in the RIP sample. (DOC 50 kb) [file 13059_2015_839_MOESM2_ESM.doc]

| **a 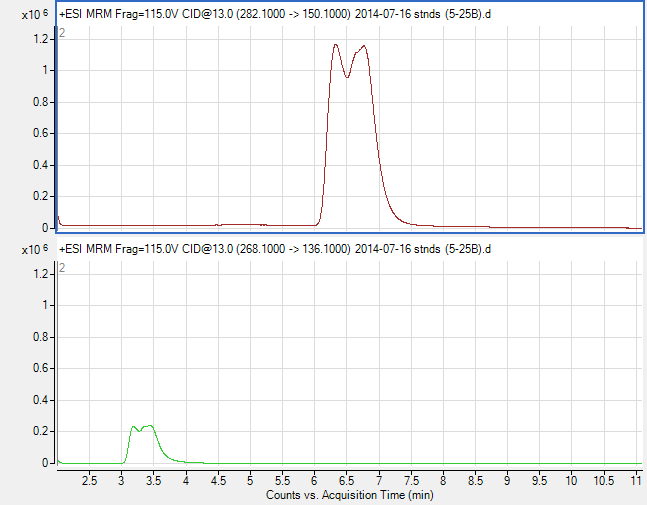** |
| --- |
| **b**  **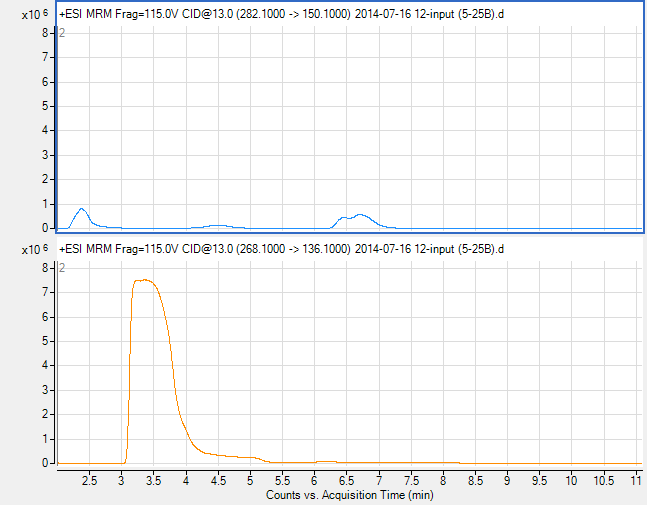** |
| **c**  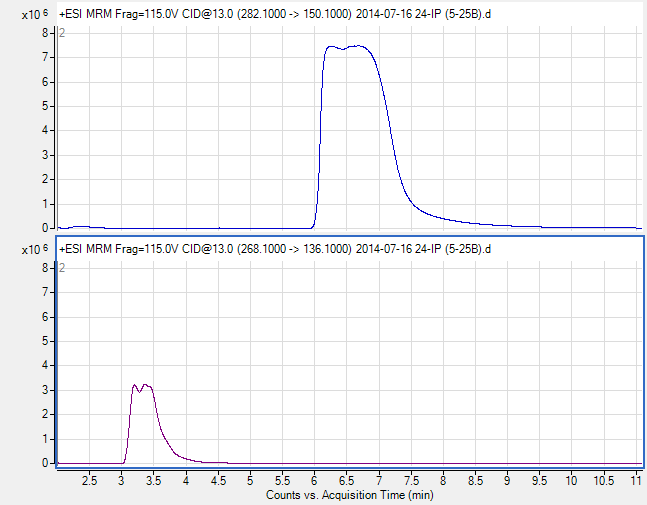 |

**Additional file 2:** **Figure S1. The m^6^A peak and adenosine peak deduced from the HPLC-MS/MS analysis.** **a** The relative m^6^A peak height (upper) and adenosine peak height (lower) in the standard sample. **b** The relative m^6^A peak height (upper) and adenosine peak height (lower) in the input sample. **c** The relative m^6^A peak height (upper) and adenosine peak height (lower) in the RIP sample.

Because ratio of m^6^A/A is much different between the IP and input samples (Fig. S1b and S1c), it is really very difficult to arrange a proper mole of m^6^A and adenosine in the standard sample suitable for estimation of the m^6^A and adenosine concentration in the both IP and input samples, indicating that the data in the Fig. S1 may not reflect the absolute concentration in the samples and can not be used for estimation of the ‘absolute’ ratio of m^6^A/A in the samples because a relative large error may occur in the HPLC-MS-MS analysis. However, we could use the HPLC-MS-MS data to estimate the non-specific immunoprecipitation rate. Ratio of m^6^A/A in the IP sample will be equal to that in the input sample supposed that the anti-body does not have any specific binding ability and ratio of m^6^A/A in the IP sample will be very close to that in the input sample in this case. Thus, ratio of m^6^A/A in the IP sample will decrease with an increase of the non-specific immunoprecipitation rate. And the non-specific immunoprecipitation rate could approximately be estimated by this formula considered that the error is very close between the input and IP samples in the HPLC-MS-MS analysis because all the samples were processed with three replicates in the same batch on the machine during the HPLC-MS-MS analysis: the non-specific immunoprecipitation rate = ratio of m^6^A/A in the input sample divided by ratio of m^6^A/A in the IP sample. The non-specific immunoprecipitation rate was mostly lower than 1% in this study using this formula.
