# Supplementary material for: Transcriptome-wide high-throughput deep m6A-seq reveals unique differential m6A methylation patterns between three organs in Arabidopsis thaliana
Source: Genome Biol. 2015 Dec 14;16:272. doi: 10.1186/s13059-015-0839-2 (PMC4714525; doi:10.1186/s13059-015-0839-2)
Supplement: Additional file 3: Table S2. — Number of transcripts in the mRNA-seq and the m6A-seq (RIP) samples and proportion of the m6A modified transcripts in the three organs of Arabidopsis. (DOC 33 kb) [file 13059_2015_839_MOESM3_ESM.doc]

**Additional file 3:** **Table S2.** **Number of transcripts in the mRNA-seq and the m^6^A-seq (RIP) samples and proportion of the m^6^A modified transcripts in the three organs of *Arabidopsis***

| Replicates |  | Leaves | Flowers | Roots |
| --- | --- | --- | --- | --- |
| Replicate 1 | RNA-seq | 24,102 | 26,599 | 24,554 |
|  | m^6^A-seq | 16,412 | 18,329 | 17,643 |
|  | Proportion (%)^a^ | 68.1 | 68.9 | 69.1 |
| Replicate 2 | RNA-seq | 23,242 | 25,880 | 23,815 |
|  | m^6^A-seq | 16,963 | 20,307 | 20,010 |
|  | Proportion (%)^a^ | 70.6 | 73.7 | 76.6 |

^a^Proportion of the transcripts containing m^6^A modification in the *Arabidopsis* transcriptome.
