# Supplementary material for: Transcriptome-wide high-throughput deep m6A-seq reveals unique differential m6A methylation patterns between three organs in Arabidopsis thaliana
Source: Genome Biol. 2015 Dec 14;16:272. doi: 10.1186/s13059-015-0839-2 (PMC4714525; doi:10.1186/s13059-015-0839-2)
Supplement: Additional file 4: Table S3. — Number of m6A sites detected in the three organs of Arabidopsis. (DOC 37 kb) [file 13059_2015_839_MOESM4_ESM.doc]

**Additional file 4:** **Table S3. Number of m^6^A sites detected in the three organs of *Arabidopsis***

| Replicates |  | Leaves | Flowers | Roots |
| --- | --- | --- | --- | --- |
| Replicate 1 | Total transcripts (n) | 24,102 | 26,599 | 24,554 |
|  | m^6^A transcripts (n) | 16,412 | 18,329 | 17,643 |
|  | Total m^6^A sites | 35,891 | 39,870 | 47,395 |
|  | m^6^A sites per m^6^A transcript | 2.2 | 2.2 | 2.7 |
|  | m^6^A sites per transcript | 1.5 | 1.5 | 1.9 |
|  | Ratio m^6^A/A (%) | 0.44 | 0.45 | 0.55 |
| Replicate 2 | Total transcripts (n) | 23,242 | 25,880 | 23,815 |
|  | m^6^A transcripts (n) | 16,963 | 20,307 | 20,010 |
|  | Total m^6^A sites | 28,803 | 47,051 | 48,884 |
|  | m^6^A sites per m^6^A transcript | 1.7 | 2.3 | 2.4 |
|  | m^6^A sites per transcript | 1.2 | 1.8 | 2.1 |
