# Supplementary material for: Transcriptome-wide high-throughput deep m6A-seq reveals unique differential m6A methylation patterns between three organs in Arabidopsis thaliana
Source: Genome Biol. 2015 Dec 14;16:272. doi: 10.1186/s13059-015-0839-2 (PMC4714525; doi:10.1186/s13059-015-0839-2)
Supplement: Additional file 5: Table S4. — Ratio of m6A/A in the three organs of Arabidopsis. (DOC 38 kb) [file 13059_2015_839_MOESM5_ESM.doc]

**Additional file 5:** **Table S4. Ratio of m^6^A/A in the three organs of *Arabidopsis***

| Replicates |  | Leaves | Flowers | Roots |
| --- | --- | --- | --- | --- |
| Replicate 1 | Total m^6^A sites | 35,891 | 39,870 | 47,395 |
|  | Total A in the modified transcripts | 8,233,861 | 8,882,747 | 8,655,860 |
|  | Total A in the transcriptome | 11,136,101 | 11,961,976 | 11,284,530 |
|  | Ratio m^6^A/A in the m^6^A transcripts | 0.44 | 0.45 | 0.55 |
|  | Ratio m^6^A/A in the transcriptome | 0.32 | 0.33 | 0.42 |
|  | Ratio m^6^A/A (%) | 0.44 | 0.45 | 0.55 |
| Replicate 2 | Total m^6^A sites | 28,803 | 47,051 | 48,884 |
|  | Total A in the modified transcripts | 6,109,367 | 8,177,627 | 7,324,231 |
|  | Total A in the transcriptome | 11,081,723 | 11,805,175 | 11,125,970 |
|  | Ratio m^6^A/A in the m^6^A transcripts | 0.47 | 0.58 | 0.67 |
|  | Ratio m^6^A/A in the transcriptome | 0.26 | 0.40 | 0.44 |
