# Supplementary material for: Transcriptome-wide high-throughput deep m6A-seq reveals unique differential m6A methylation patterns between three organs in Arabidopsis thaliana
Source: Genome Biol. 2015 Dec 14;16:272. doi: 10.1186/s13059-015-0839-2 (PMC4714525; doi:10.1186/s13059-015-0839-2)
Supplement: Additional file 6: Table S5. — Category of the m6A modified transcripts based on the number of m6A sites per transcript. (DOC 37 kb) [file 13059_2015_839_MOESM6_ESM.doc]

**Additional file 6:** **Table S5.** **Category of the modified transcripts based on the number of m^6^A sites per transcript**

| Replicates | Plant organs | Proportion of transcripts with different m^6^A sites (%) | | | | | |
| --- | --- | --- | --- | --- | --- | --- | --- |
|  |  | 1 site | 2 sites | 3 sites | 4 sites | 5 sites | >5 sites |
| Replicate 1 | Leaf | 53.8 | 17.6 | 11.0 | 7.0 | 3.7 | 6.9 |
|  | Flower | 48.7 | 21.4 | 11.7 | 7.6 | 4.1 | 6.5 |
|  | Root | 45.7 | 18.7 | 11.5 | 5.3 | 5.1 | 13.7 |
|  | Average | 49.4 | 19.2 | 11.4 | 6.6 | 4.3 | 9.0 |
| Replicate 2 | Leaf | 54.8 | 15.5 | 8.9 | 6.0 | 4.0 | 11.0 |
|  | Flower | 56.5 | 17.1 | 11.5 | 8.4 | 5.7 | 13.2 |
|  | Root | 42.6 | 15.7 | 9.1 | 7.8 | 5.4 | 19.5 |
|  | Average | 51.3 | 16.1 | 9.8 | 7.4 | 5.0 | 14.5 |
