# Supplementary material for: Transcriptome-wide high-throughput deep m6A-seq reveals unique differential m6A methylation patterns between three organs in Arabidopsis thaliana
Source: Genome Biol. 2015 Dec 14;16:272. doi: 10.1186/s13059-015-0839-2 (PMC4714525; doi:10.1186/s13059-015-0839-2)
Supplement: Additional file 7: Table S6. — Proportion of two types of m6A distributing feature in mRNA. (DOC 30 kb) [file 13059_2015_839_MOESM7_ESM.doc]

**Additional file 7: Table S6. Proportion of two types of m^6^**A distributing feature in mRNA

|  | Type 1 (%) | | | Type 2 (%) | | |
| --- | --- | --- | --- | --- | --- | --- |
|  | Leaves | Flowers | Roots | Leaves | Flowers | Roots |
| Replicate 1 | 76.2 | 74.0 | 75.1 | 23.8 | 26.0 | 24.9 |
| Replicate 2 | 76.4 | 71.9 | 74.6 | 23.6 | 28.1 | 25.3 |

Type 1, dominant m^6^A enrichment was observed in stop codon or 3'UTR. Accordingly, dominant m^6^A enrichment was not observed in stop codon or 3'UTR in Type 2.
