# Supplementary material for: Transcriptome-wide high-throughput deep m6A-seq reveals unique differential m6A methylation patterns between three organs in Arabidopsis thaliana
Source: Genome Biol. 2015 Dec 14;16:272. doi: 10.1186/s13059-015-0839-2 (PMC4714525; doi:10.1186/s13059-015-0839-2)
Supplement: Additional file 8: Table S7. — The transcriptome-wide normalized read depth in the 60 bins of the gene representing the overall m6A patterns in the different regions of the genes. Non-significant differences were found between three organs (P = 0.761). (DOC 108 kb) [file 13059_2015_839_MOESM8_ESM.doc]

**Additional file 8: Table S7. The transcriptome-wide normalized read depth in the 60 bins of the gene representing the overall m^6^A patterns in the different regions of the genes.**

| Bins | Replicate 1 | | | Replicate 2 | | |
| --- | --- | --- | --- | --- | --- | --- |
|  | Leaf_ | Flower_ | Root_ | Leaf | Flower | Root |
| 0 | 1.573864 | 1.80105 | 2.248538 | 3.358909 | 3.065202 | 2.89864 |
| 1 | 2.805262 | 3.15739 | 3.901977 | 6.724471 | 6.632674 | 6.009696 |
| 2 | 3.423169 | 3.579506 | 4.531052 | 8.776943 | 8.779243 | 7.986502 |
| 3 | 3.565826 | 3.372795 | 4.290879 | 9.720624 | 9.794371 | 8.945909 |
| 4 | 3.607474 | 2.806274 | 4.158391 | 9.93144 | 10.12269 | 9.195627 |
| 5 | 3.92039 | 3.635474 | 4.415505 | 9.751014 | 10.10976 | 9.185187 |
| 6 | 4.096948 | 3.647693 | 4.208492 | 9.568922 | 10.08913 | 9.165795 |
| 7 | 3.962866 | 3.473876 | 3.9449 | 9.242763 | 9.736999 | 9.023545 |
| 8 | 3.878847 | 3.30432 | 4.023244 | 9.133121 | 9.376774 | 8.920397 |
| 9 | 3.863539 | 3.46968 | 4.137809 | 9.142036 | 9.247475 | 8.931121 |
| 10 | 3.762628 | 3.400236 | 4.03913 | 8.876123 | 8.904283 | 8.774975 |
| 11 | 3.71686 | 3.332971 | 3.935892 | 8.670099 | 8.567719 | 8.595997 |
| 12 | 3.900408 | 3.331179 | 3.979963 | 8.463196 | 8.309589 | 8.615238 |
| 13 | 3.830587 | 3.291565 | 3.852807 | 8.361144 | 8.275112 | 8.692295 |
| 14 | 3.535271 | 3.106056 | 3.672495 | 8.271594 | 8.201981 | 8.622893 |
| 15 | 3.147638 | 2.629321 | 3.189018 | 8.196986 | 8.126411 | 8.698404 |
| 16 | 3.275981 | 2.896686 | 3.648593 | 8.287057 | 8.158191 | 8.80557 |
| 17 | 3.403188 | 3.367518 | 3.884898 | 8.281893 | 8.141525 | 8.883496 |
| 18 | 3.492098 | 3.391142 | 3.867631 | 8.435455 | 8.207771 | 8.874498 |
| 19 | 3.387234 | 3.318154 | 3.735488 | 8.514483 | 8.310613 | 9.054888 |
| 20 | 3.166034 | 3.163499 | 3.753171 | 8.539248 | 8.281023 | 9.027894 |
| 21 | 3.200758 | 3.439267 | 3.98939 | 8.402861 | 8.261889 | 9.150451 |
| 22 | 3.294948 | 3.322794 | 3.917824 | 8.385269 | 8.290356 | 9.155425 |
| 23 | 3.180565 | 3.284922 | 3.866882 | 8.236128 | 8.32121 | 9.133005 |
| 24 | 3.254853 | 3.365864 | 3.980171 | 8.352438 | 8.264685 | 9.288097 |
| 25 | 3.21274 | 3.464705 | 3.925319 | 8.509646 | 8.34912 | 9.32882 |
| 26 | 3.307131 | 3.375982 | 3.868286 | 8.360043 | 8.35499 | 9.233159 |
| 27 | 3.335249 | 3.327864 | 3.965504 | 8.299068 | 8.254128 | 9.347656 |
| 28 | 3.330706 | 3.44492 | 4.047386 | 8.581843 | 8.395874 | 9.557663 |
| 29 | 3.3991 | 3.46029 | 4.00223 | 8.595208 | 8.57337 | 9.649219 |
| 30 | 3.499164 | 3.453621 | 3.967983 | 8.714792 | 8.755682 | 9.75847 |
| 31 | 3.767089 | 3.710213 | 4.153221 | 8.901409 | 9.035453 | 9.938889 |
| 32 | 3.956734 | 3.783205 | 4.26967 | 8.724094 | 9.059054 | 9.854838 |
| 33 | 3.912804 | 3.71866 | 4.257041 | 8.770633 | 8.969455 | 9.820038 |
| 34 | 3.949587 | 3.649008 | 4.186167 | 9.023255 | 9.237077 | 10.06154 |
| 35 | 4.102853 | 3.71729 | 4.23281 | 9.368732 | 9.36169 | 10.42403 |
| 36 | 4.257062 | 3.768432 | 4.324589 | 9.43505 | 9.569354 | 10.67568 |
| 37 | 4.287431 | 3.673976 | 4.163182 | 9.538665 | 9.640476 | 10.65135 |
| 38 | 4.470933 | 3.656978 | 4.425261 | 9.910589 | 9.809773 | 10.9349 |
| 39 | 4.516176 | 3.880556 | 4.499466 | 9.804161 | 9.782729 | 10.98939 |
| 40 | 4.630558 | 4.00749 | 4.632732 | 9.950922 | 10.10521 | 11.24819 |
| 41 | 4.736275 | 3.836655 | 4.607128 | 9.927794 | 10.3043 | 11.31511 |
| 42 | 4.546973 | 3.605855 | 4.536093 | 10.0594 | 10.43593 | 11.52134 |
| 43 | 4.364061 | 3.4518 | 4.42735 | 10.08887 | 10.59107 | 11.60157 |
| 44 | 4.580112 | 3.770901 | 4.740083 | 10.20777 | 10.82581 | 11.76007 |
| 45 | 4.941576 | 4.337386 | 4.949345 | 10.25403 | 11.11694 | 11.86442 |
| 46 | 5.381063 | 4.653373 | 5.189815 | 10.3576 | 11.38195 | 12.05825 |
| 47 | 5.875139 | 5.06859 | 5.636012 | 10.45009 | 11.67752 | 12.24509 |
| 48 | 6.294266 | 5.672147 | 6.151504 | 10.58864 | 11.89418 | 12.53223 |
| 49 | 7.055041 | 6.459959 | 6.714569 | 10.77624 | 12.52983 | 13.02618 |
| 50 | 8.213529 | 7.808196 | 7.742817 | 11.41701 | 13.72978 | 14.03207 |
| 51 | 10.06637 | 9.690432 | 8.99342 | 11.90618 | 14.85506 | 14.69398 |
| 52 | 13.20389 | 12.65936 | 11.13075 | 12.39112 | 16.31175 | 15.86885 |
| 53 | 17.5801 | 16.47752 | 14.0277 | 13.36969 | 18.23356 | 17.59262 |
| 54 | 23.37685 | 21.52451 | 17.6242 | 14.69077 | 20.3343 | 19.46437 |
| 55 | 31.54497 | 29.31086 | 24.64074 | 16.65137 | 23.41449 | 22.54697 |
| 56 | 42.65628 | 40.43284 | 34.48545 | 18.35283 | 26.63102 | 26.15133 |
| 57 | 48.1495 | 48.84505 | 42.63431 | 19.38844 | 28.00903 | 29.30317 |
| 58 | 44.97341 | 48.57779 | 45.3797 | 17.11072 | 24.32764 | 28.07067 |
| 59 | 23.58088 | 26.80212 | 26.66121 | 9.097402 | 12.63731 | 16.19758 |
