# Supplementary material for: Transcriptome-wide high-throughput deep m6A-seq reveals unique differential m6A methylation patterns between three organs in Arabidopsis thaliana
Source: Genome Biol. 2015 Dec 14;16:272. doi: 10.1186/s13059-015-0839-2 (PMC4714525; doi:10.1186/s13059-015-0839-2)
Supplement: Additional file 9: Table S8. — The primers used for qRT-PCR. (DOC 35 kb) [file 13059_2015_839_MOESM9_ESM.doc]

**Additional file 9: Table S8.** The primers used for qRT-PCR

| Gene ID | Forward primers | Reverse primers |
| --- | --- | --- |
| At1G35710 | CACCAGAGTTTGCTTACACG | TCGAGTATCAAAACCCCGAAG |
| At3g07610 | CTCGAACACATATCTTCAAGCG | TTTTCTCCGCTCATATGTCCTC |
| At4g14410 | TCGATCAAAGCAATGGAGCA | TTTGCAGACTGATCAAGAGGATACCTTTCC |
| At1g03880 | GCCCACTTTCTTGAACGC | TTGACCTTCTCCAAATACCGG |
| At2g28490 | GTATACCTCGTAAATCTCACCGC | CTCTCCAGAGCCAGCTAAAAC |
| At3g13400 | CCATTTGGACGGTTACTCCTTC | GCATCGAGCAAGTTGTAGTTG |
| At1g33700 | GCTCCTGTTCTTGCTGACACAAC | AGTGGTATAGAGTAACCCTCTTCA |
| At2g07835 | GAAAGTTGTGGTTGTCCTCTTC | CAATCCGTTTGTCTTTGCCTC |
| At4g38120 | AATGTGAATTGACTGAGCAAGC | GTCACTTGAGTTGGAAATTACAGG |
| At5g20960 | GGAGATTCTCAACAGTGGACAAC | GTGAACAGAAGCCGCTAAAAG |
| At5g22700 | GCTGTCCAAATCTCAAATCCC | ATGATAGGAAACACGGAGGC |
| AT3G18780-*Actin2* | TCGCCATCCAAGCTGTTC | ACACCATCACCAGAATCCAG |
