# Supplementary material for: Transcriptome-wide high-throughput deep m6A-seq reveals unique differential m6A methylation patterns between three organs in Arabidopsis thaliana
Source: Genome Biol. 2015 Dec 14;16:272. doi: 10.1186/s13059-015-0839-2 (PMC4714525; doi:10.1186/s13059-015-0839-2)
Supplement: Additional file 10: Figure S2. — The relative abundance (RA) of m6A RNA deduced from qRT-PCR and the expected abundance (EA) of m6A RNA deduced from the m6A-seq dataset. a RA for ‘AT1G35710’, b EA for ‘AT1G35710’, c RA for ‘AT3G07610’, d EA for ‘AT3G07610’, e RA for ‘AT4G14410’, f EA for ‘AT4G14410’, g RA for ‘AT2G28490’, h EA for ‘AT2G28490’, i RA for ‘AT1G03880’, j RA for ‘AT1G03880’, k RA for ‘AT1G33700’, l EA for ‘AT1G33700’, m RA for ‘AT2G07836’, n EA for ‘AT2G07836’, o RA for ‘AT5G20960’, p EA for ‘AT5G20960’, q RA for ‘AT5G22700’, r EA for ‘AT5G22700’, s RA for ‘AT3G13400’, t EA for ‘AT3G13400’, u EA for ‘AT4G38120’, v EA for ‘AT4G38120’. (DOC 1342 kb) [file 13059_2015_839_MOESM10_ESM.doc]

| **a**  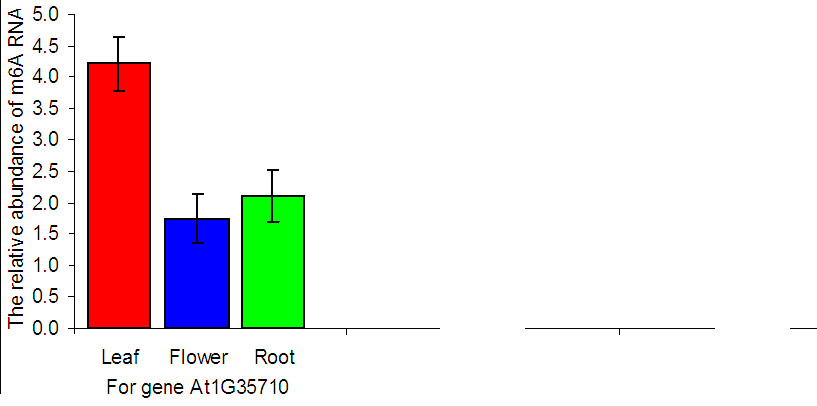 | **b**  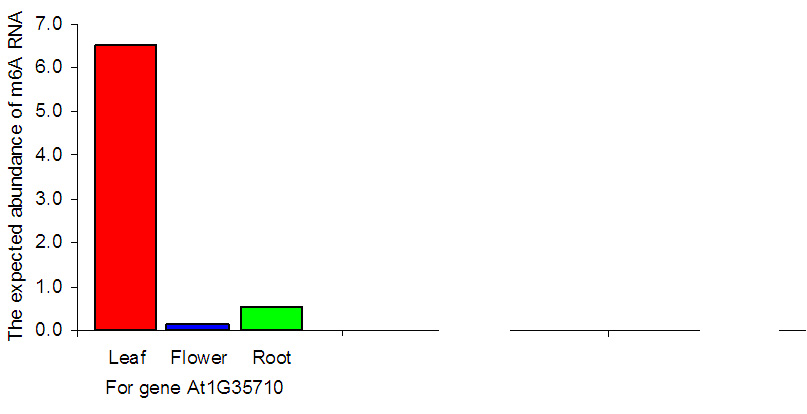 | **c**  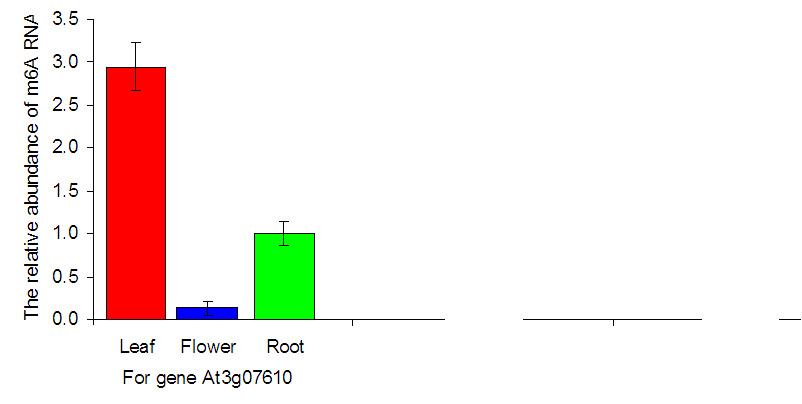 | **d**  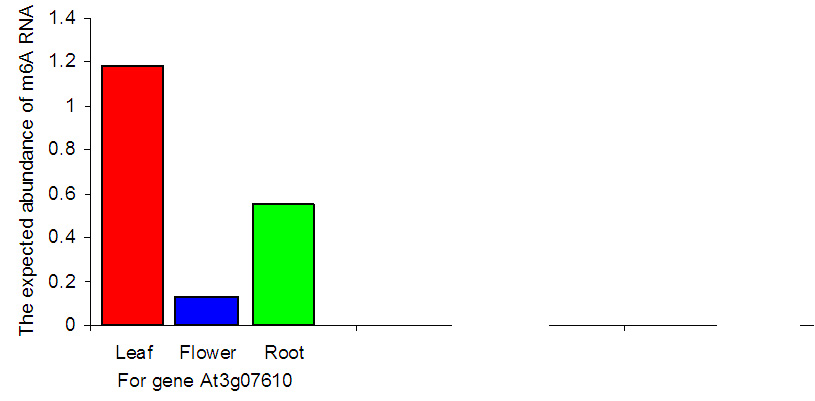 |
| --- | --- | --- | --- |
| **e**  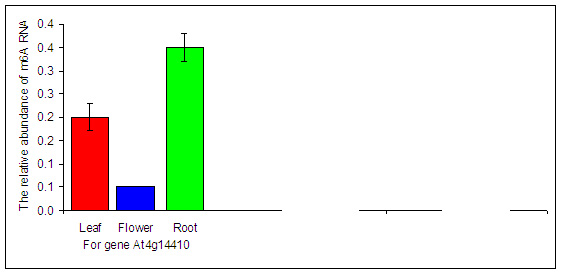 | **f**  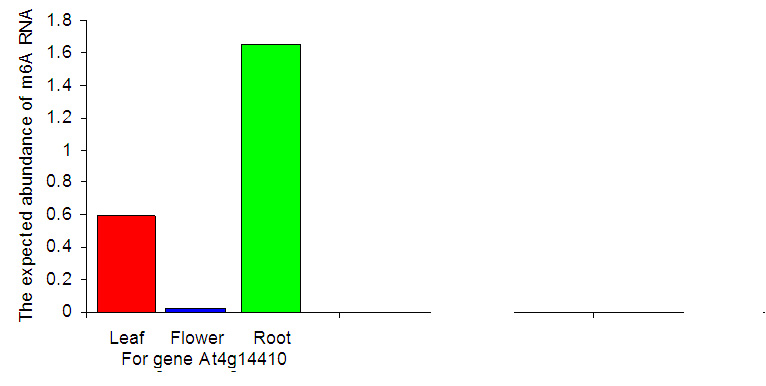 | **g**  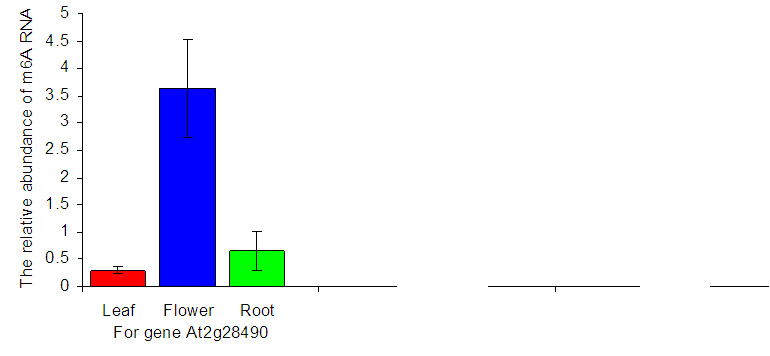 | **h**  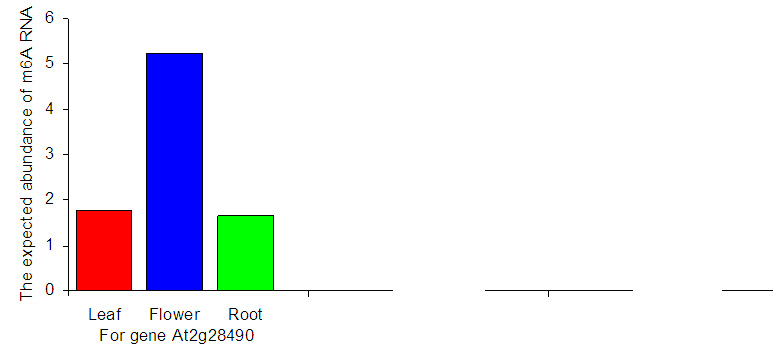 |
| **i**  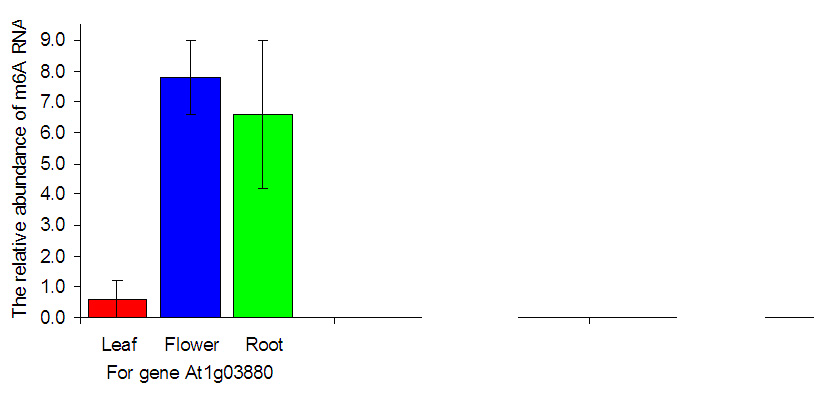 | **j**  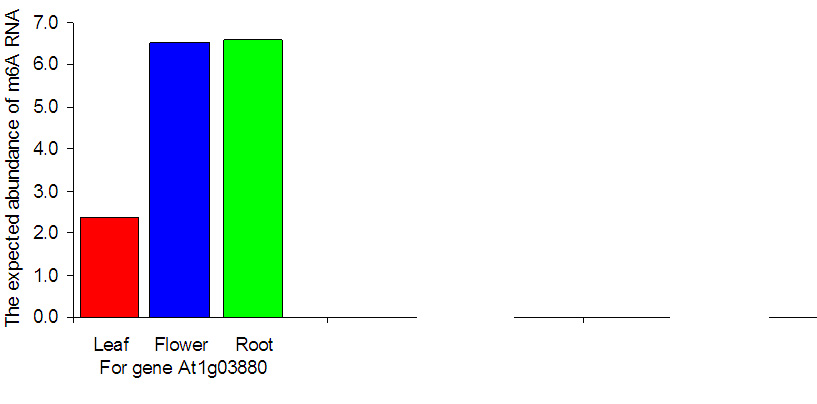 | **k**  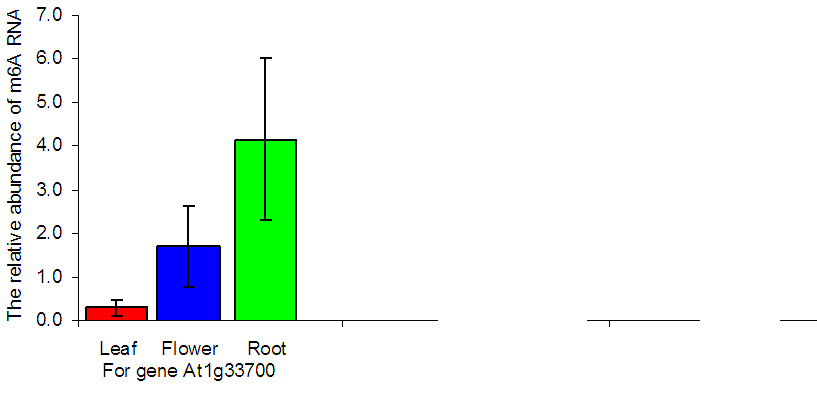 | **l**  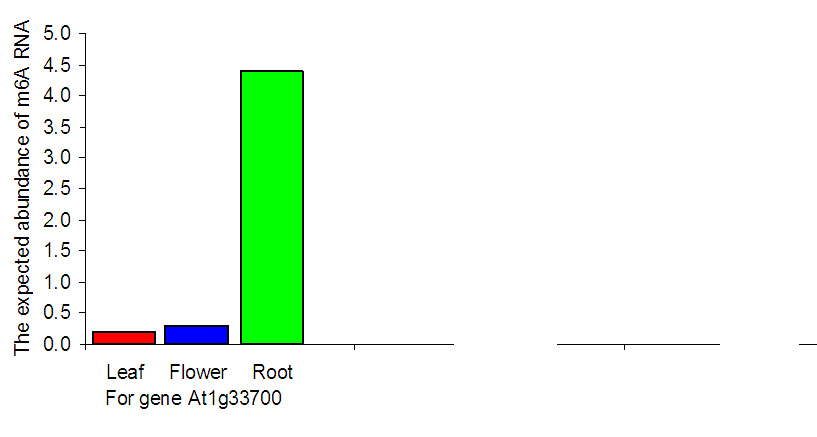 |

| **m**  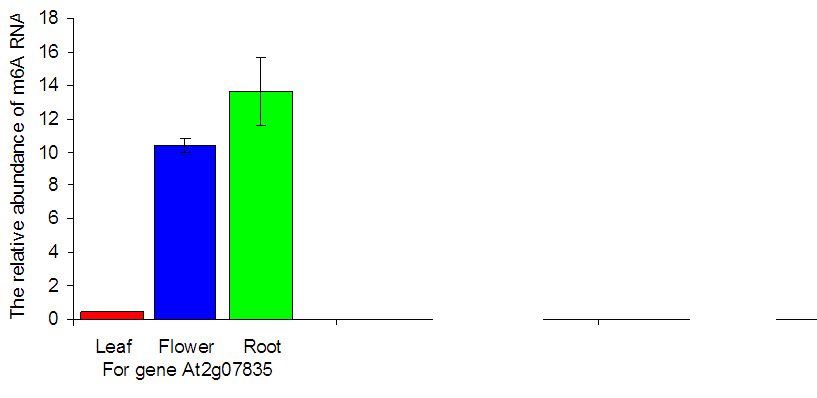 | **n**  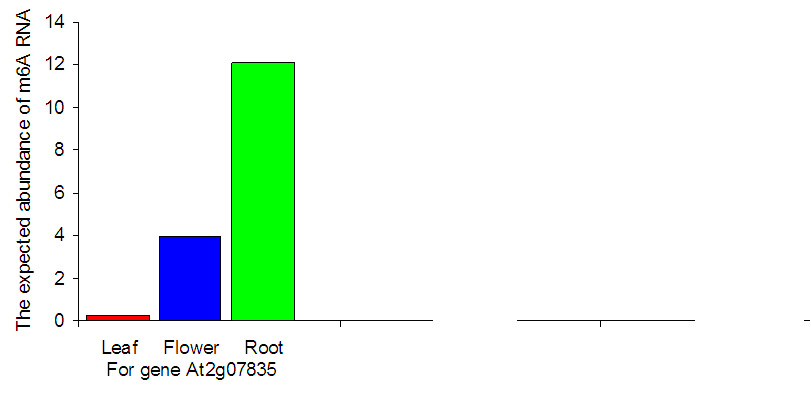 | **o**  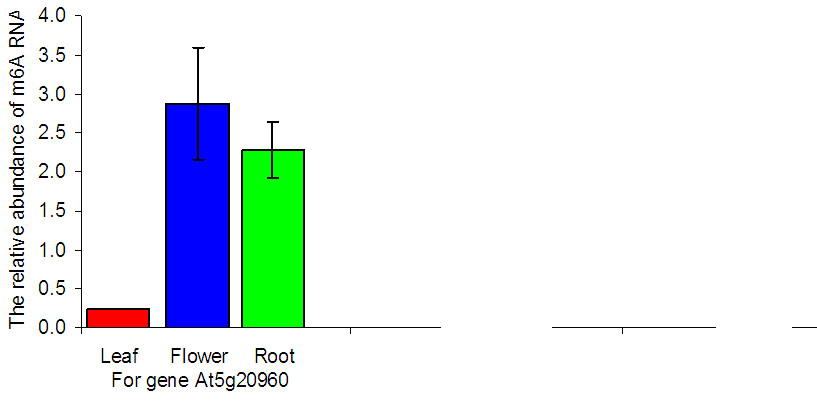 | **p**  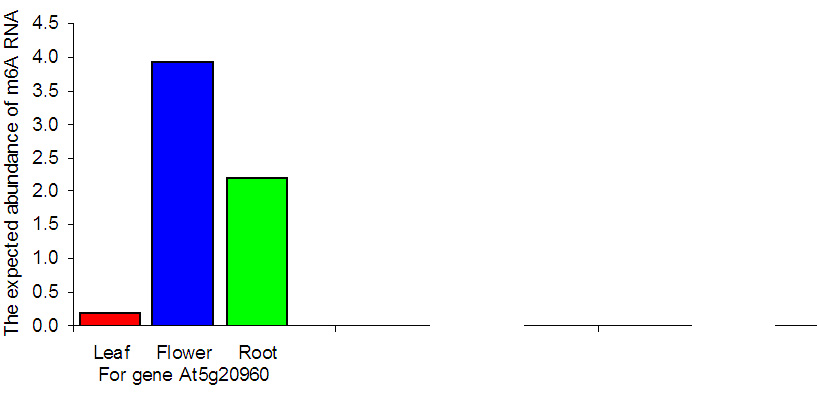 |
| --- | --- | --- | --- |
| **q**  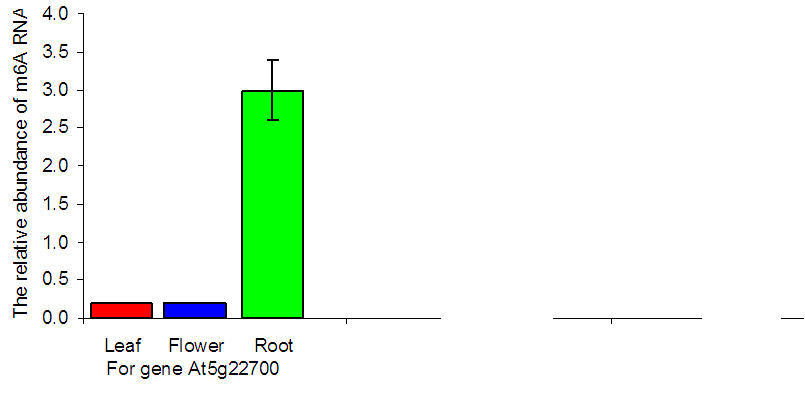 | **r**  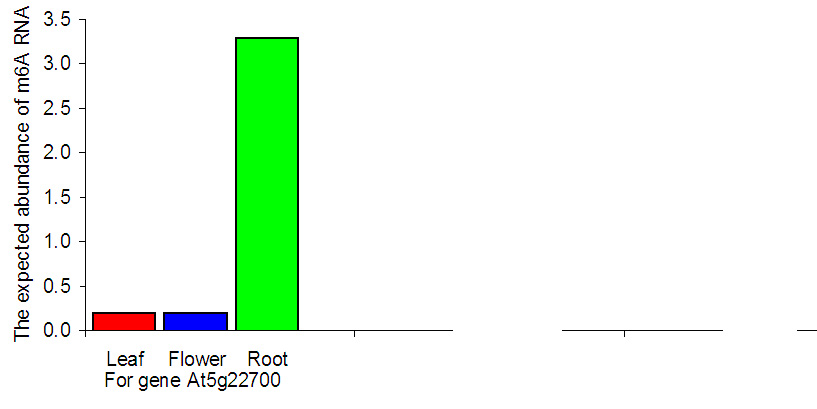 | **s**  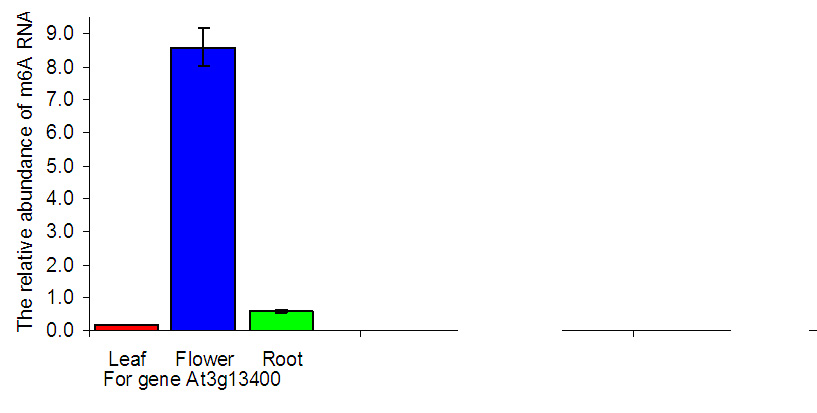 | **t**  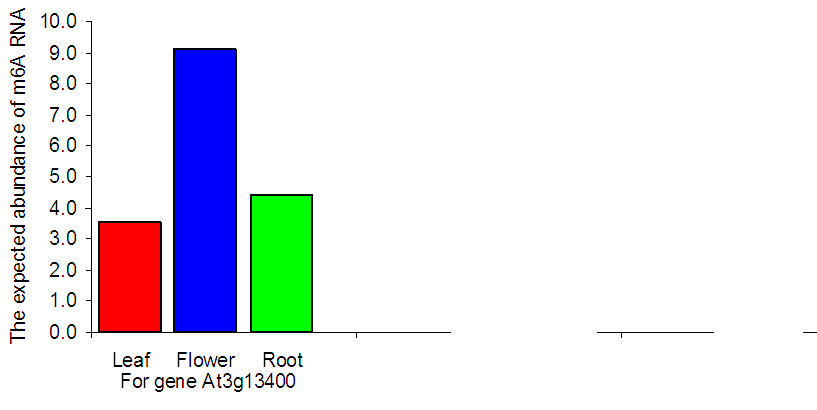 |
| **u**  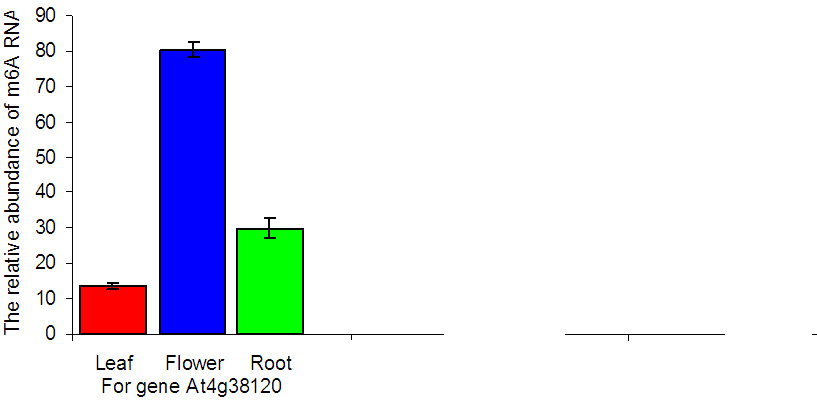 | **v**  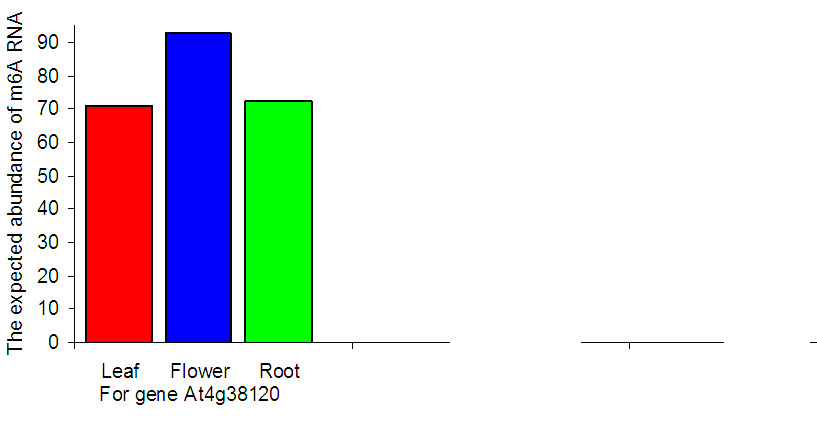 |  |  |

**Additional file 10:** **Figure S2. The relative abundance (RA) of m^6^A RNA deduced from qRT-PCR and the expected abundance (EA) of m^6^A RNA deduced from the m^6^A-seq data set.** **a** RA for ‘AT1G35710’, **b** EA for ‘AT1G35710’, **c** RA for ‘AT3G07610’, **d** EA for ‘AT3G07610’, **e** RA for ‘AT4G14410’, **f** EA for ‘AT4G14410’, **g** RA for ‘AT2G28490’, **h** EA for ‘AT2G28490’, **i** RA for ‘AT1G03880’, **j** RA for ‘AT1G03880’, **k** RA for ‘AT1G33700’, **l** EA for ‘AT1G33700’, **m** RA for ‘AT2G07836’, **n** EA for ‘AT2G07836’, **o** RA for ‘AT5G20960’, **p** EA for ‘AT5G20960’, **q** RA for ‘AT5G22700’, **r** EA for ‘AT5G22700’, **s** RA for ‘AT3G13400’, **t** EA for ‘AT3G13400’, **u** EA for ‘AT4G38120’, **v** EA for ‘AT4G38120’.
