# Supplementary material for: Transcriptome-wide high-throughput deep m6A-seq reveals unique differential m6A methylation patterns between three organs in Arabidopsis thaliana
Source: Genome Biol. 2015 Dec 14;16:272. doi: 10.1186/s13059-015-0839-2 (PMC4714525; doi:10.1186/s13059-015-0839-2)
Supplement: Additional file 11: Figure S3. — RNA QC results of the total RNA and the RIP RNA for m6A-seq samples. a RNA quality for the total RNA sample was high with RIN over 8.5. b RNA fragmentation for the m6A-seq samples was consistent in the experiments, with an average length of 106 nt. (DOC 313 kb) [file 13059_2015_839_MOESM11_ESM.doc]

| **a For total RNA** | **b For RIP RNA sample** |
| --- | --- |
| **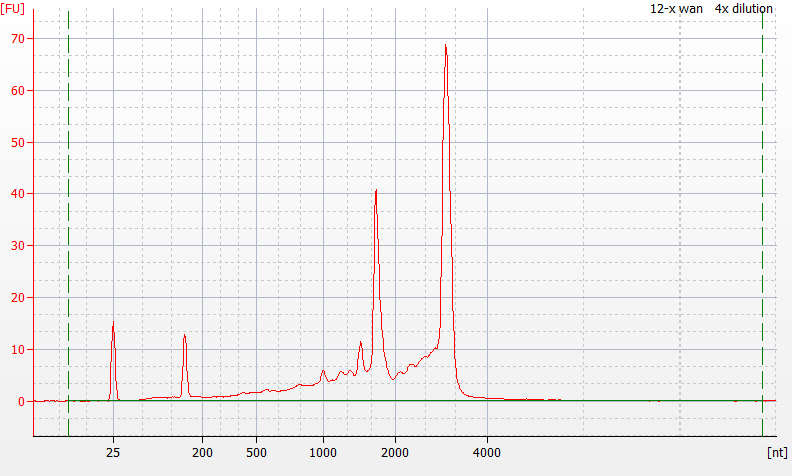** | 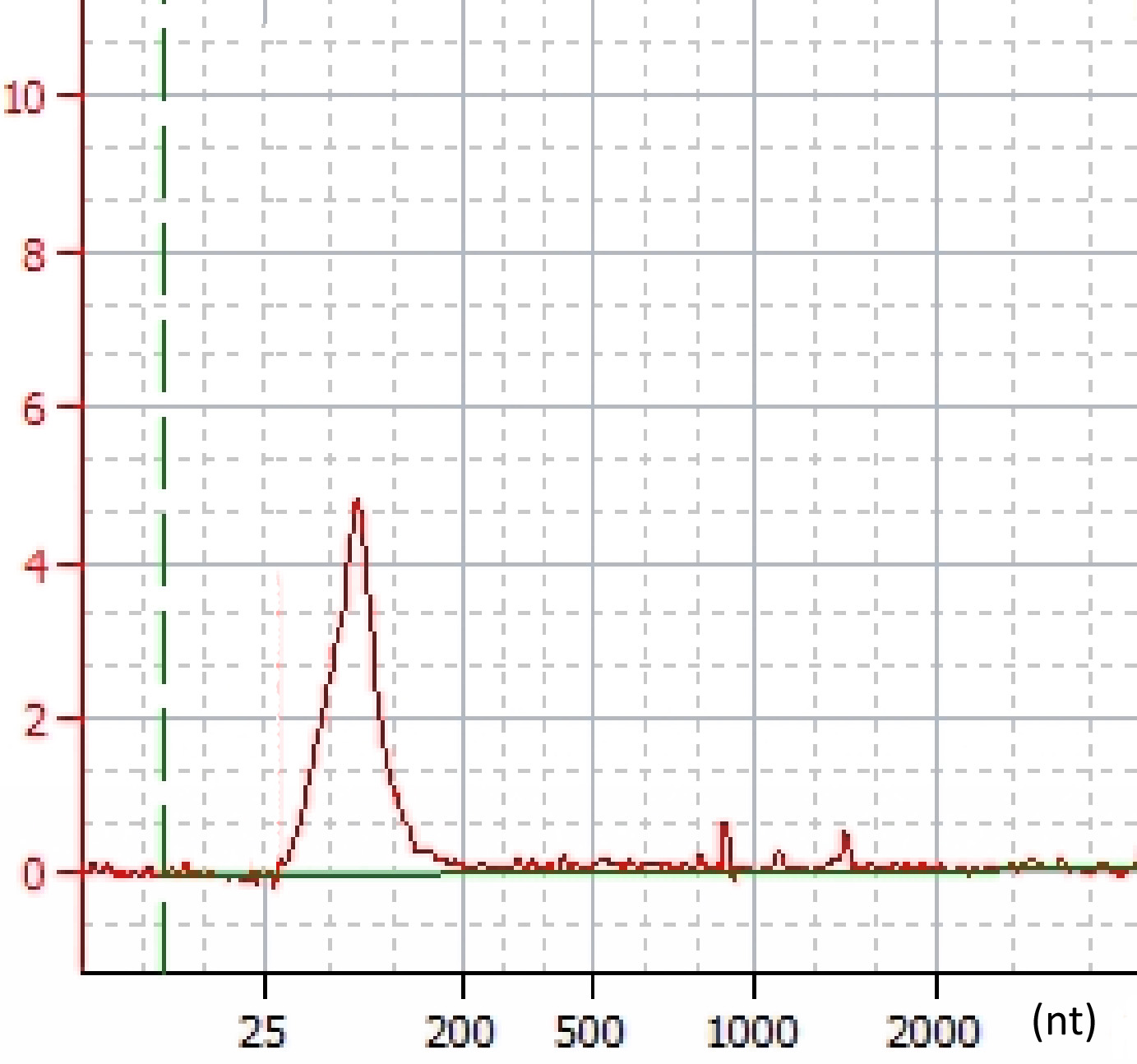 |

**Additional file 11:** **Figure S3. RNA QC results of the total RNA and the RIP RNA for m^6^A-seq samples.** **a** RNA quality for the total sample was high with RIN over 8.5. **b** RNA fragmentation for the m^6^A-seq samples was consistent in the RIP experiments, with an average length of 106 nt.
